# A framework for the regional critical zone classification: the case of the Chinese Loess Plateau

**DOI:** 10.1093/nsr/nwy147

**Published:** 2018-11-26

**Authors:** Yihe Lü, Jian Hu, Bojie Fu, Paul Harris, Lianhai Wu, Xiaolin Tong, Yingfei Bai, Alexis J Comber

**Affiliations:** 1 State Key Laboratory of Urban and Regional Ecology, Research Center for Eco-Environmental Sciences, Chinese Academy of Sciences, China; 2 Joint Center for Global Change Studies, China; 3 University of Chinese Academy of Sciences, China; 4 Institute of Qinghai-Tibetan Plateau, Southwest Minzu University, China; 5 Sustainable Agriculture Sciences, Rothamsted Research, UK; 6 The Grain for Green Project Management Office of Yan'an Municipality, China; 7 Leeds Institute for Data Analytics and School of Geography, University of Leeds, UK

The concept of the Earth's Critical Zone (CZ)—the near-surface heterogeneous environment of our planet—was originally defined to include the land surface, vegetation canopy, rivers, lakes and shallow seas [1]. CZ accommodates interactions among air, water, soil, rock and living organisms, and determines the availability of life-sustaining resources needed for the well-being and sustainability of human society. Therefore, there are new opportunities for integrative studies of the CZ as a key research frontier [1,2] to address the grand challenges of global sustainability in the twenty-first century [3].

In CZ research, attention is given to processes operating from the vegetation canopy to rock in the vertical dimension. Studies of CZ structure, processes, functions and evolution provide the core scientific themes in contemporary CZ science [e.g. 4–7]. Consequently, natural laboratories and field-based observations with integrated modeling were advocated at the outset as key methodological tools for addressing these themes, along with multidisciplinary collaborations, particularly across local to regional scales [1,8].

To date, progress in CZ science has strengthened our understanding of the responses of the near-surface processes to climatic and human perturbations. This has been underpinned by the establishment of CZ observatories and their networks [9]. Monitoring-based research activities have grown significantly due to the development and operation of the observatories. Therefore, most of their findings are restricted to local scales on structures, processes and their interactions. A more comprehensive picture of CZ structures, processes and functions at watershed, regional and global scales can be derived by establishing networks of CZ observatories that facilitate statistical inference [10]. To aid such an expansion, some studies have investigated the spatial heterogeneity of key CZ characteristics in large watersheds and across regional scales through the analysis of long-distance transect survey data or of regionally distributed watersheds of different sizes along environmental gradients [11–13]. At the global scale, CZ thickness and its controlling factors have been quantified by combining climate, vegetation height, water-table depth, groundwater thickness, topography and lithological data [14]. Nevertheless, a comprehensive typology of CZs at regional scales is still lacking and there has been insufficient development of a classification methodology to do this [15]. Such a CZ classification could provide a cornerstone for the cost-effective prioritization and planning of CZ observatories and, in doing so, advance CZ science.

To address this research gap, we provide an operational framework for classifying CZ types at the regional scale (Fig. 1). According to the underpinning concepts of CZs, a CZ can be characterized by its geological, biological, ecological and atmospheric features, and human and socioeconomic factors. In our framework, we use the term ‘geodiversity' to refer to the structural diversity of CZs within a specific geographic region. It can be quantified by geological, geomorphological, soil, hydrological and topographical properties of the CZ [16–18]. Climate operates as a driver that modifies not only Earth-surface conditions, but also the distributions of biota [16,18]. Therefore, we considered geodiversity, ecosystems and climate as the three key features of CZ. However, humans have exerted huge impacts on CZs through demands for food, materials and living spaces. Hence, we also included human and socioeconomic factors as anthropogenic driving forces of CZ change.

**Figure 1.**
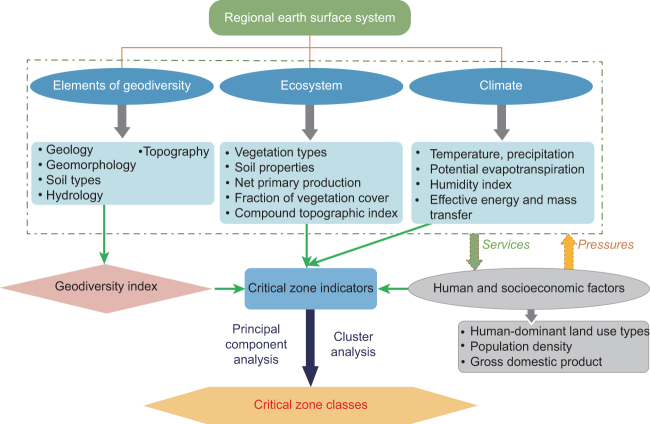
The operational framework for classifying the types of CZs at the regional scale.

## REGIONAL CZ CLASSIFICATION

The Chinese Loess Plateau (CLP) is the largest and deepest loess deposit area in the world and is the most successful ecological restoration zone in China (Supplementary Figs 1–3, available as Supplementary Data at *NSR* online). In our operational framework, 24 CZ indicators were obtained from spatial datasets and used to classify the CLP (Supplementary Table 1 and Figs 7–10, available as Supplementary Data at *NSR* online). This was achieved by transforming the 24 indicator variables through a principal components analysis (PCA) and using the PC scores from first six PCs as inputs into a clustering algorithm (details for indicators and methods can be found in the Supplementary Data, available at *NSR* online).

**Figure 2.**
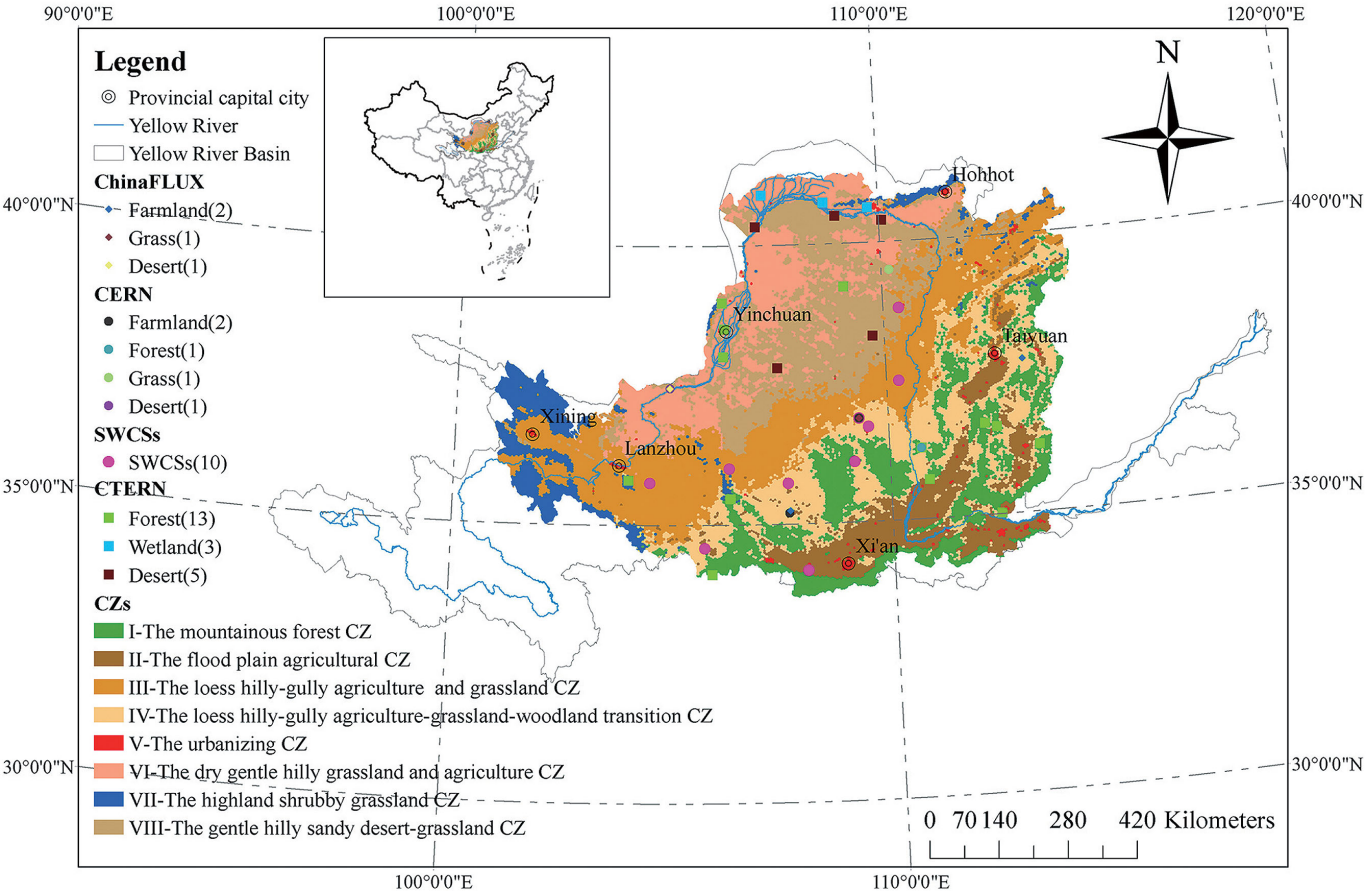
The types of CZs and field-observation stations of the CLP region. ChinaFLUX, Chinese FLUX Observation and Research Network; CERN, Chinese Ecosystem Research Network; CTERN, Chinese Terrestrial Ecosystem Research Network; SWCSs, Soil and Water Conservation Stations.

The resulting eight CZ classes were optimally determined by evaluating the within-group sum of squares (low values) and pseudo F-statistics (high values) of different numbers of clusters (Supplementary Figs 12 and 13, available as Supplementary Data at *NSR* online). The distribution and percentage of each CZ class are mapped in Fig. 2 and plotted in Supplementary Fig. 14, available as Supplementary Data at *NSR* online. A nomenclature was applied to the eight classes, with the principal aim of reflecting the geographical characteristics of the class, its vegetation as well as auxiliary factors, such as soil and climate. The characteristics and heterogeneity of the eight CZ classes in terms of their geodiversity, terrain, climate, energy, vegetation, soil properties, and human and socioeconomic indicators are shown using error-bar plots (Supplementary Figs 15–20, available as Supplementary Data at *NSR* online).

There are clear differences between the eight CZ categories in their typical positions in the indicator feature space used for the classification. Class I is termed ‘mountainous forest' CZ and is found in 13.11% of CLP, with trees and shrubs accounting for 49.92 and 31.29% of the area, respectively. Class II is termed the ‘floodplain agricultural' CZ. It accounts for 9.75% of the CLP, with cropland covering over 60% of its area, the highest of the eight CZ classes. Class III is termed the ‘loess hill-gully agriculture and grassland' CZ, covering 22.51% of the CLP, with a mean cropland and grassland coverage of 43.92

and 43.20%, respectively. Class IV is termed the ‘loess hill-gully agriculture-grassland-woodland transition' CZ, with agriculture having a mean percentage cover of 34.78%, grassland having a mean percentage cover of 38.96% and woodland having a mean percentage cover of 23.95% (second in percentage cover in the CLP). Class III and Class IV CZs are typical of a loess region, with higher geodiversity than the other six CZ classes (Supplementary Figs 3 and 15, available as Supplementary Data at *NSR* online). In addition, these two CZ classes represent the most significant vegetation recovery regions in China after the implementation of the national government's sloping cropland re-vegetation program known as ‘Grain for Green' in 1999 [19].

Class V is the smallest CZ class and is termed the ‘urbanizing' CZ (accounting for 0.78% of the CLP). This class had the highest Gross Domestic Product and population density, at 13470.95 10^4^ yuan/km^2^ and 4917.17 Individuals/km^2^, respectively. The spatial distribution of the ‘urbanizing' CZ class is relatively patchy and spatially interacts with all other CZ classes (Fig. 2). Class VI is termed the ‘dry gentle hilly grassland and agriculture' CZ, with mean grassland and cropland coverage of 50.15 and 24.80%, respectively. Class VII is termed the ‘highland shrubby grassland' CZ, with mean grass and shrub coverage of 42.75 and 28.88%, respectively. The final class, class VIII, is termed the ‘gentle hilly sandy desert-grassland' CZ, with a grassland percentage coverage of 54.74%, but with 15.03% of the region reclaimed as cropland.

The above CZ classification is an integrative one that incorporates multiple indicators, which themselves are representative of key biophysical properties, socioeconomic characteristics, land surface conditions and deep geological features (depth of loess soil and rocks). This is novel and unlike many ecological or physical geography-based regionalization studies [20–22]. However, the framework and its indicators were not exhaustive and can be adapted according to the situations of other regions.

## THE IMPORTANCE OF CAPTURING SPATIAL HETEROGENEITIES

CZ science is in its second decade of development [15]. There are still challenges associated with further advancement related to the highly dynamic and heterogeneous nature of CZs, which require interdisciplinary and integrated approaches [10]. One of the toughest challenges involves deep-coupling research, which seeks to link across different spatiotemporal scales, across different CZ components and their interactions. In order to address this challenge, there is a need to build a network of CZ observatories that traverse the regional CZ types, and to do this in a scientifically informed and cost-effective manner.

Characterizing the spatial variation in regional CZs can provide insight for the prioritization and systematic planning of CZ observatories. There are always trade-offs between the number of field-based observatories and measurement detail.

For example, a site-scale CZ observatory in the USA (the Susquehanna Shale Hills Critical Zone Observatory) has been enlarged from its original 0.08-km^2^ catchment to a 164-km^2^ watershed to accommodate the wider spatial processes, including lithologies and land uses [10]. However, most field-based studies have avoided taking a ‘everything and everywhere' measurement philosophy and instead have focused on measuring only those features necessary to study the local CZ as a holistic Earth-surface system. Therefore, the problem of determining how many field-based observatories are needed and deciding on the scope and detail of relevant measurements have been barriers for the advancement of CZ science, especially from data acquisition and methodological development perspectives at regional scales.

To address these problems, the regional geographical classification of CZ systems can be used to inform the design of sampling frameworks to cover different types of CZs with certain spatial configurations specific to different regional contexts. At least one formal CZ observatory is needed for each CZ class and a common biophysical-based measurement scheme is required. This should be formulated to characterize key entities, including atmosphere, water, biota, regolith and land surface [10,23] and their interactions across all CZ observatories in a given region. Optional measurements in the human dimension that are relevant to each CZ class can be used to provide information about the socioeconomic services supported by CZs [24,25].

The results of this study (Fig. 2) exemplifies the potential for trade-offs in prioritization and systematic planning of CZ observatories and measurements. To date, a series of field-based observatories have been established in the CLP region by different organizations, such as the Chinese FLUX Observation and Research Network (ChinaFLUX), the Chinese Ecosystem Research Network (CERN) and Soil and Water Conservation Stations (SWCSs) (Fig. 2). Most of these observatories are characterized by the dominant ecosystem types or Earth-surface processes. To establish a network of CZ observatories in the CLP region, a practical and effective approach would be to update and adapt existing observatories according to the requirements of integrated CZ science [4,5,9,10,24] and then to bridge any gaps by establishing new observatories in CZ classes lacking observatories. In this manner, a cost-efficient regional CZ observatory network can be planned and established in the CLP. From this study's results, the CZ classes I, II, V and VII are under-represented in the existing observatory network (Fig. 2) and should be prioritized in future CZ observatory planning and construction. This approach for developing functioning CZ observatory networks is adaptable to other regions and at continental and global scales [26].

Besides improving CZ observations, recognizing the regional variation of CZs can result in improved understanding and modeling of horizontal CZ interactions. In an interconnected and increasingly globalizing world, the scientific investigation of CZs should not be confined only to few specific locations or to very local scales, as observed CZ changes at one location can result from changes in the CZs elsewhere. CZ drivers and impacts over geographical distances have recently been recognized as ‘telecoupling' in ecological and environmental research [26–28]. The CZ is intrinsically affected by local couplings and telecouplings from both biophysical and socioeconomic respects. The categorization of regional CZs provides a spatially explicit framework for considering such couplings as well as supporting hypothesis testing and model development [29].

In the CLP region, the ‘urbanizing' CZs (Class V) intersect with all the other CZ classes (Fig. 2). There are close links in environmental impacts and flows of materials and ecosystem services among urban and other CZs [30,31]. Similarly, investigation of the trans-boundary processes and services of CZ classes I and VII (Fig. 2), predominantly in the highlands and mountainous areas, would advance understanding of the functional links (e.g. hydrological links) between CZs and support regional conservation (e.g. soil and habitat conservation) and development planning. In summary, regional-scale, integrative understandings of the spatial interactions among different types of CZs on processes, functions and services are key for the advancement of CZ science as a core interdisciplinary and transdisciplinary research field for targeting and underpinning environmental sustainability [24].

## Supplementary Material

Supplemental File
