# Supplementary material for: A framework for the regional critical zone classification: the case of the Chinese Loess Plateau
Source: Natl Sci Rev. 2018 Nov 26;6(1):14–8. doi: 10.1093/nsr/nwy147 (PMC8291574; doi:10.1093/nsr/nwy147)
Supplement: Supplemental File [file nwy147_supplemental_file.doc]

**Supplementary Data**

**This file includes:**

⦁ **Supplementary Text** describing the study area of the Chinese Loess Plateau (CLP), data preparation for the critical zone (CZ) indicators, and statistical outputs.

⦁ **Supplementary Fig. S1.** Spatial distribution of basic landforms in the CLP.

⦁ **Supplementary Fig. S2.** Spatial distribution of geological age (A), rock types (B) and Quaternary sediments (C) in the CLP. (Q: Quaternary; NQ: Neogene-Quaternary; N: Neogene; PN: Palaeogene-Neogene; PE: Palaeogene; CP: Cretaceous-Palaeogene; C: Cretaceous; JC: Jurassic and Cretaceous; J: Jurassic; TJ: Trias-Jurassic; T: Trias; PT: Permian-Trias; UP: Upper Palaeozoic; P: Permian; CP: Carboniferous-Permian; CB: Carboniferous; DC: Devonian-Carboniferous; D: Devonian; LP: Lower Paleozoic; SLR: Silurian; O: Ordovician; EO: Cambrian-Ordovician; E: Cambrian; N: Neoproterozoic; SN: Sinian; NS: Nanhuan-Sinian; QBK: Qingbaikouan; MP: Mesoproterozoic; JX: Jixian; CCG: Changchengian; PM: Palaeoproterozoic-Mesoproterozoic; PP: Palaeoproterozoic; HT: Hutuo; NAP: New Archaeozoic-Palaeoproterozoic; A: Archaeozoic; NA: New Archaean; PM: Paleoarchean-Mesoarchean; MA: Mesoarchean; PA: Paleoarchean).

⦁ **Supplementary Fig. S3.** Spatial distribution of loess types and depth in the CLP.

⦁ **Supplementary Fig. S4.** Distribution of components in terms of geological age (A), lithology (B), slope position (C), landform categories (D), soil types (E) and hydrology (F).

⦁ **Supplementary Fig. S5.** The richness of geological age (A), lithology (B), slope position (C), landform (D), soil types (E) and hydrology (F).

⦁ **Supplementary Fig. S6.** Total geodiversity (A) and roughness (B) of the CLP.

⦁ **Supplementary Fig. S7.** Geodiversity index (GI) of the CLP.

⦁ **Supplementary Fig. S8.** The CZ indicators from ecosystems, soil particles, and topography including fraction of vegetation cover (A), net primary production (B), ratios of shrub (C), ratios of grass (D), ratios of tree (E), clay (F), gravel (G), organic carbon (H), sand (I), silt (J), soil bulk density (K), and compound topographic index (L).

⦁ **Supplementary Fig. S9.** The CZ indicators from the climate including annual mean precipitation (A), annual mean temperature (B), potential evapotranspiration (C), humidity index (D), net primary production energy and mass transfer (E), heat energy related to effective precipitation energy and mass transfer (F) and effective energy and mass transfer (G).

⦁ **Supplementary Fig. S10.** The CZ indicators from perspectives of human pressures and socioeconomic factors, including the ratio of artificial ground (A), the ratio of farmland (B), gross domestic product (C) and population density (D).

⦁ **Supplementary Fig. S11.** PCA scree plot with complementary analyses.

⦁ **Supplementary Fig. S12.** Within-group sum of squares with the *k*-means cluster analysis.

⦁ **Supplementary Fig. S13.** Pseudo F-statistic from the *k*-means cluster analysis.

⦁ **Supplementary Fig. S14.** The percentage area of each CZ class in the CLP. The sample sizes (n) of Classes I, II, III, IV, V, VI, VII and VIII are 3870, 2878, 6648, 4868, 229, 4526, 1704 and 4804, respectively.

⦁ **Supplementary Fig. S15.** Geodiversity index (GI) and compound topographic index (CTI) for the eight CZ types in the CLP. The sample size (n) of Classes I, II, III, IV, V, VI, VII and VIII are 3870, 2878, 6648, 4868, 229, 4526, 1704 and 4804, respectively.

⦁ **Supplementary Fig. S16.** Climate indicators for the eight CZ classes in the CLP. The sample size (n) of Classes I, II, III, IV, V, VI, VII and VIII are 3870, 2878, 6648, 4868, 229, 4526, 1704 and 4804, respectively.

⦁ **Supplementary Fig. S17.** Effective energy and mass transfer indicators for the eight CZ classes in the CLP. The sample size (n) of Classes I, II, III, IV, V, VI, VII and VIII are 3870, 2878, 6648, 4868, 229, 4526, 1704 and 4804, respectively.

⦁ **Supplementary Fig. S18.** Vegetation indicators for the eight CZ classes in the CLP. The sample size (n) of Classes I, II, III, IV, V, VI, VII and VIII are 3870, 2878, 6648, 4868, 229, 4526, 1704 and 4804, respectively.

⦁ **Supplementary Fig. S19.** Soil properties for the eight CZ classes in the CLP. The sample size (n) of Classes I, II, III, IV, V, VI, VII and VIII are 3870, 2878, 6648, 4868, 229, 4526, 1704 and 4804, respectively.

⦁ **Supplementary Fig. S20.** Human and socioeconomic indicators for the eight CZ classes in the CLP. The sample size (n) of Classes I, II, III, IV, V, VI, VII and VIII are 3870, 2878, 6648, 4868, 229, 4526, 1704 and 4804, respectively.

⦁ **Supplementary Table S1.** Datasets used for the classification of the CZs in the CLP.

⦁ **Supplementary Table S2.** Fundamental components of geodiversity.

⦁ **Supplementary Table S3.** Landform classes based on topographic position index and slope degree.

⦁ **Supplementary Table S34.** Pressure surrogates using human dominant land use types.

⦁ **Supplementary Table S5.** Summary statistics of the 24 CZ indicators in the CLP.

⦁ **Supplementary Table S6.** Pearson correlation coefficients for the 24 CZ indicators in the CLP.

⦁ **Supplementary Table S7.** The contribution of variances explained by the first six principal components.

⦁ **Supplementary Table S8.** PCA loadings of the CZ indicators for the first six components.

**The Chinese Loess Plateau (CLP) as a key area for critical zone (CZ) research**

The CLP covers about 640,000 km^2^. According to the national basic geomorphology, the CLP contains plain, platform, hill and mountain landforms (Supplementary Fig. S1). In addition, the region has a typical loess geomorphology including loess Yuan, Liang, and Mao with various gullies of different erosion magnitudes [1, 2]. The geological age of most of the region is mainly in the Quaternary, and Quaternary sediments were widely distributed (Supplementary Fig. S2 (A) and (B)). Loess, fluvial, pluvial, alluvial and lake deposits were the main Quaternary deposit types (Supplementary Fig. S2 (C)). The CLP is both the largest and the deepest loess deposit area in the world. The loessial soil is the dominant loess type with loess depth commonly greater than 50m, and in some areas over 250m (Supplementary Fig. S3). The soils in the region have been characterized as globally the most highly erodible [3], where many soil and water conservation measures have been implemented since the 1970s [1] after intensive agricultural reclamation in the 1950s-1960s for crop production. With the implementation of the Grain-for-Green Project (re-vegetation on the sloping croplands) since 1999, the CLP has become the most successful ecological restoration zone in China [4]. Details about loess soil, geological, geomorphological, vegetation, climate characteristics, and the change of land use and some earth surface processes in the CLP have also been synthetically reported [2]. As a whole, the CLP has a heterogeneous and dynamic environment that sustains about 8.5% of the Chinese population with only 6.6% of the national land area with a population density of 168 people per square kilometer. Therefore, the CLP is an ideal place for CZ research from perspectives of evolution, structure, process, function and service of CZ systems around the globe. In particular, it provides a typical case study for research into the spatial heterogeneity and the spatial interactions linked to human-CZ relationships.


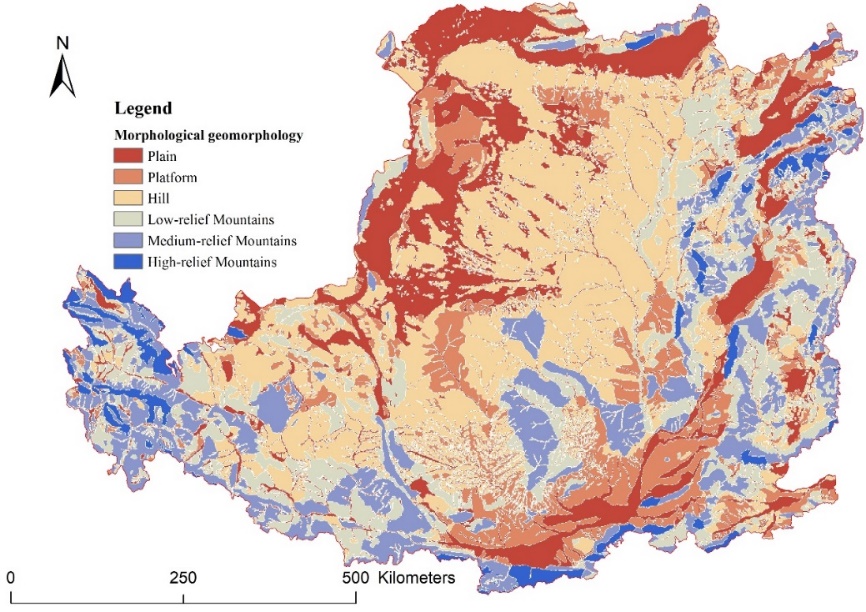


**Supplementary Fig. S1.** Spatial distribution of basic landforms in the CLP (Modified from 100M China Geomorphology Map, 2009).


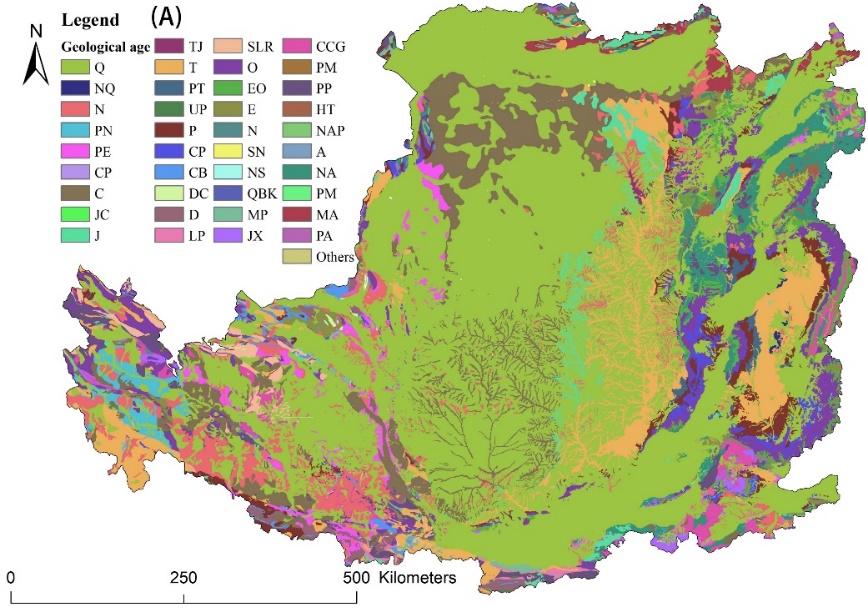


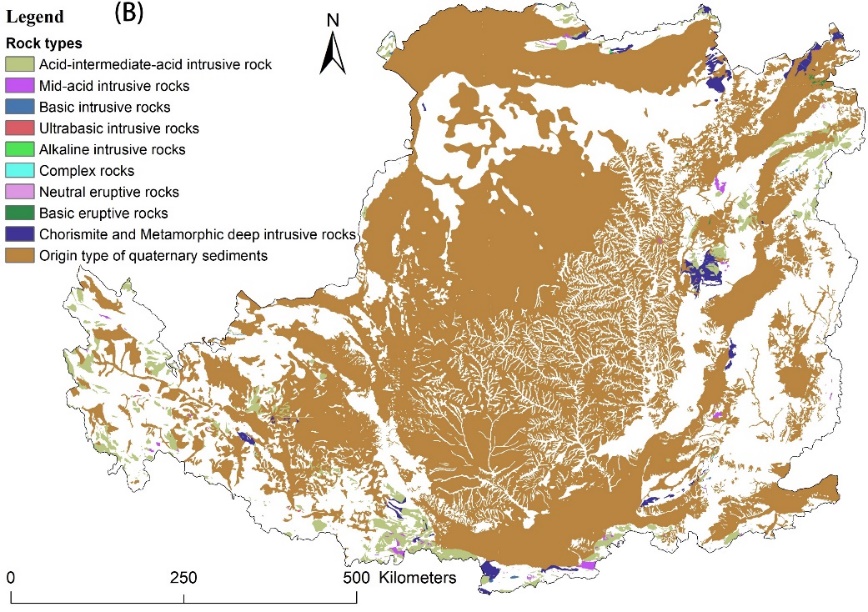


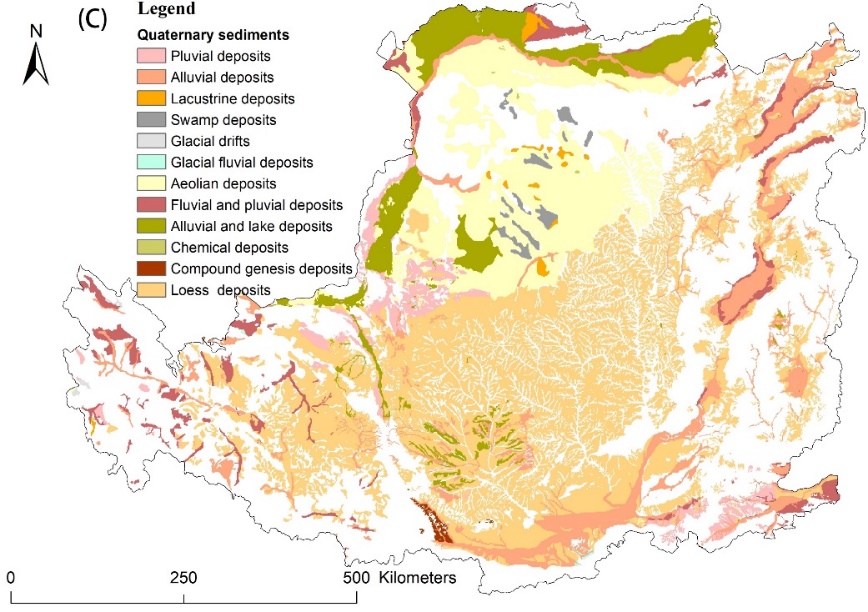


**Supplementary Fig. S2.** Spatial distribution of geological age (A), rock types (B) and Quaternary sediments (C) in the CLP. (Q: Quaternary; NQ: Neogene-Quaternary; N: Neogene; PN: Palaeogene-Neogene; PE: Palaeogene; CP: Cretaceous-Palaeogene; C: Cretaceous; JC: Jurassic and Cretaceous; J: Jurassic; TJ: Trias-Jurassic; T: Trias; PT: Permian-Trias; UP: Upper Palaeozoic; P: Permian; CP: Carboniferous-Permian; CB: Carboniferous; DC: Devonian-Carboniferous; D: Devonian; LP: Lower Paleozoic; SLR: Silurian; O: Ordovician; EO: Cambrian-Ordovician; E: Cambrian ; N: Neoproterozoic; SN: Sinian; NS: Nanhuan-Sinian; QBK: Qingbaikouan; MP: Mesoproterozoic; JX: Jixian; CCG: Changchengian; PM: Palaeoproterozoic-Mesoproterozoic; PP: Palaeoproterozoic; HT: Hutuo; NAP: New Archaeozoic-Palaeoproterozoic; A: Archaeozoic; NA: New archaean; PM: Paleoarchean-Mesoarchean; MA: Mesoarchean; PA: Paleoarchean).


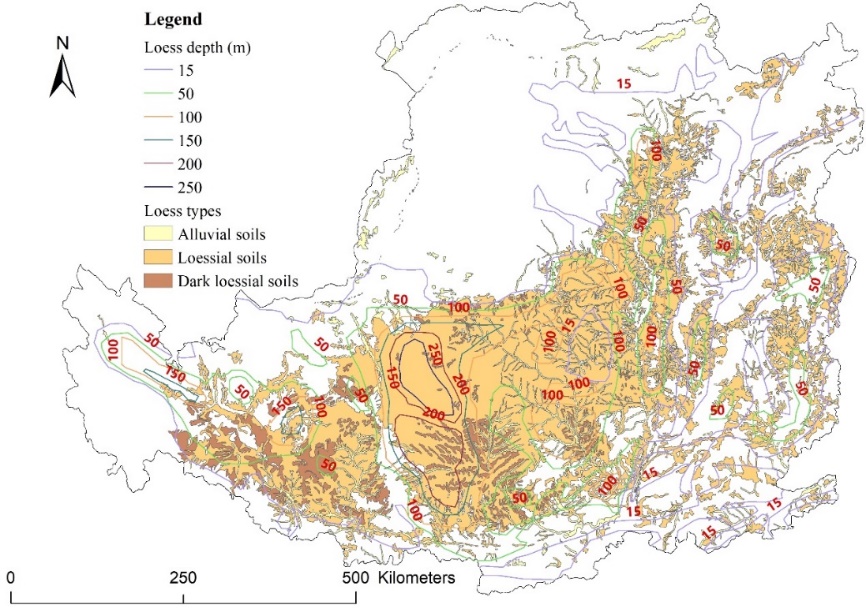


**Supplementary Fig. S3.** Spatial distribution of loess types and depth in the CLP.

**Data processing and calculation of CZ indicators**

We collected multi-source data describing the spatial patterns of geological, terrain, climate, soil, vegetation, hydrology and human-social-economic factors. These were used to formulate and derive 24 CZ indicators in the CLP (Supplementary Table S1).

**Supplementary Table S1.** Datasets used for the regionalization of the CLP’s CZ.

| Categories | Description | Provider |
| --- | --- | --- |
| Geology | 1:1M scale China geological map | China Geological Survey (http://geodata.ngac.cn/Document/Map.aspx?MapId=EC7E1A7A7AD81954E0430100007F182E) |
| Soil | 1:1M scale China soil types map  1:1M scale China soil properties map | Institute of Soil Science, Chinese Academy of Sciences and The Ministry of Agriculture of the People's Republic of China  Environmental and Ecological Science Foundation of China  (http://westdc.westgis.ac.cn) |
| Climate | Daily meteorological data from 1980 to 2015 | Meteorological Data Center, China Meteorological Administration (<http://data.cma.cn/data/cdcindex/cid/6d1b5efbdcbf9a58.html>) |
| Net primary production | 250 m resolution net primary production from 2000 to 2015 | Institute of Remote Sensing and Digital Earth, Chinese Academy of Science |
| Fraction of vegetation cover | 250 m resolution fraction of vegetation cover from 2000 to 2015 | Institute of Remote Sensing and Digital Earth, Chinese Academy of Science |
| Land cover/landuse | 30 m resolution land use map in 2010 | Institute of Remote Sensing and Digital Earth, Chinese Academy of Science |
| Digital elevation model | 30 m resolution digital elevation model | Institute of Remote Sensing and Digital Earth, Chinese Academy of Science |
| Hydrology | 1:100M national fundamental geographic map | China Geological Survey |
| Gross domestic product | 1 km resolution national gross domestic product in 2010 | Data Center for Resources and Environmental Sciences, Chinese Academy of Sciences (RESDC) (http://www.resdc.cn) |
| Population density | 1 km resolution national population density in 2010 | Data Center for Resources and Environmental Sciences, Chinese Academy of Sciences (RESDC) (http://www.resdc.cn) |

**Geodiversity**

According to the concept of geodiversity, elements of geology, geomorphology, soil, hydrology and topography are important aspects to distinguish and assess the variety of non-biological parts on the Earth’s surface [5, 6]. Evaluation methods of geodiversity include landscape metric-based methods [7, 8], the richness of abiotic elements [9, 10] and geodiversity index methods [5, 6, 11-16]. Geodiversity, climate diversity and biodiversity support a variety of ecosystem and CZ functions and services together [17-19]. In this study, we used total geodiversity and the common geodiversity index method to assess the diversity of the non-biological components of the CZ, considering the geological, geomorphological, soil, hydrological and topographical aspects. The abiotic elements adopted in our framework can be found in Supplementary Table S2 and the spatial distributions are mapped in Supplementary Fig. S4.

**Supplementary Table S2. Fundamental components of geodiversity.**

| Types | Subtypes |
| --- | --- |
| Geology | Geological age, Lithology |
|  |  |
| Geomorphology | Slope position, Landform categories |
|  |  |
| Soil | Soil types |
| Hydrology | Rivers, Lakes, Springs |

The database of the 1:1 M Geological Map of China was used to obtain the geological richness of the CLP. The database was built by MapGIS [20]. Firstly, the vector geological map of China was converted to common E00 format by MapGIS and then to shapefile format in ArcGIS 10.0. Next, the geological layer of the CLP was extracted from this shapefile geological map of China in ArcGIS 10.0. The classification of stratigraphy was mainly based on chronostratigraphy (geological age) and yet referred to as lithostratigraphy (lithology). So, we used geological age and lithology to calculate the geological age richness and lithology richness, respectively. The geological richness was summed between geological age richness and lithology richness. The resultant shapeﬁle was converted to grid format with a cell size of 30 m. Then, the diversity of the values was computed to obtain an integer grid in a raster format where a terrain value was assigned to each pixel. We included slope position and landform categories as main components in the geomorphology by the Topographic Position Index (TPI) method [21, 22]. TPI is useful for identifying landscape patterns and boundaries that may correspond to rock type, dominant geomorphic processes, soil characteristics, vegetation, or water drainage [23]. TPI was computed by considering a 100 m radius circular neighborhood with the slope position divided into six classes including valley, lower slope, ﬂat slope, middle slope, upper slope and ridge. Comparing TPIs obtained at two different scales (500 m and 1000 m) derives the landform categories where the detected landforms are grouped into ten classes according to rules used by Weiss’s method [24]. These ten landform classes can be found in Supplementary Table S3.

**Supplementary Table S3. Landform classes based on topographic position index and slope degree.**

| Number | Morphological units | Classification rules by Weiss’s methods |
| --- | --- | --- |
| 1 | Canyons, deeply incised streams | tpi500_stdi <= -100 and tpi1000_stdi <= -100 |
| 2 | Midslope drainages, shallow valleys | tpi500_stdi <= -100 and tpi1000_stdi > -100 and tpi1000_stdi < 100 |
| 3 | Upland drainages, headwaters | tpi500_stdi <= -100 and tpi1000_stdi >= 100 |
| 4 | U-shaped valleys | tpi500_stdi > -100 and tpi500_stdi < 100 and tpi1000_stdi <= -100 |
| 5 | Plains | tpi500_stdi > -100 and tpi500_stdi < 100 and tpi1000_stdi > -100 and tpi1000_stdi < 100 and slope degree <= 5 |
| 6 | Open slopes | tpi500_std > -100 and tpi500_stdi < 100 and tpi1000_stdi > -100 and tpi1000_stdi < 100 and slope degree >= 6 |
| 7 | Upper slopes, mesas | tpi500_stdi" > -100 and tpi500_stdi < 100 and tpi1000_stdi >= 100 |
| 8 | Local ridges, hills in valleys | tpi500_stdi >= 100 and tpi1000_stdi <= -100 |
| 9 | Midslope ridges, small hills in plains | tpi500_stdi >= 100 and tpi1000_stdi > -100 and tpi1000_stdi < 100 |
| 10 | Mountain tops, high ridges | tpi500_stdi >= 100 and tpi1000_stdi >= 100 |

Note: tpi500_stdi and tpi1000_stdi were the standardization of TPI at 2 scales (500 m and 1000 m) by its mean and standard deviation.


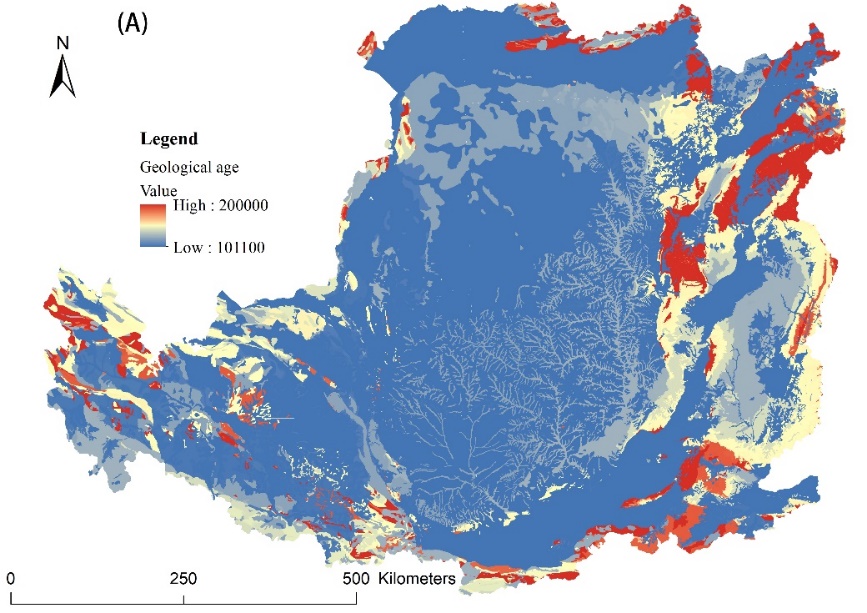


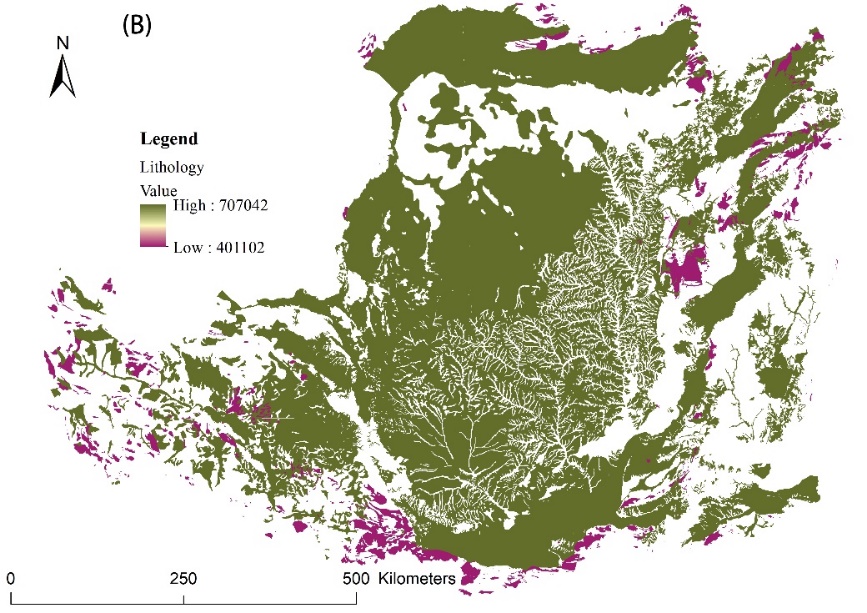


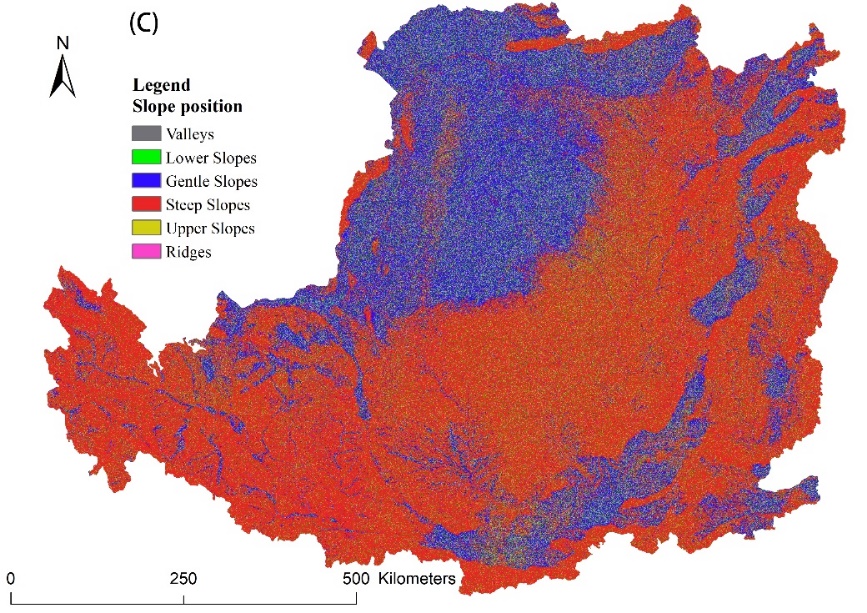


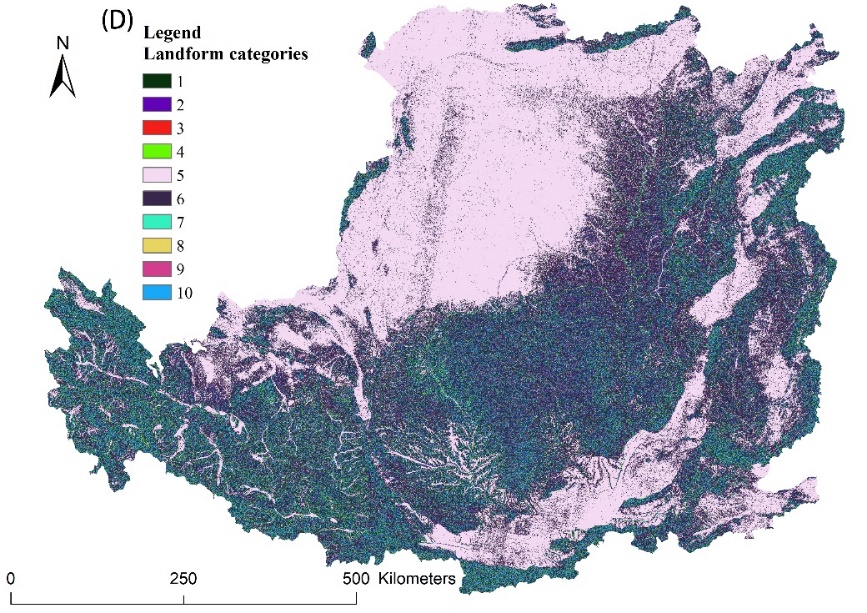


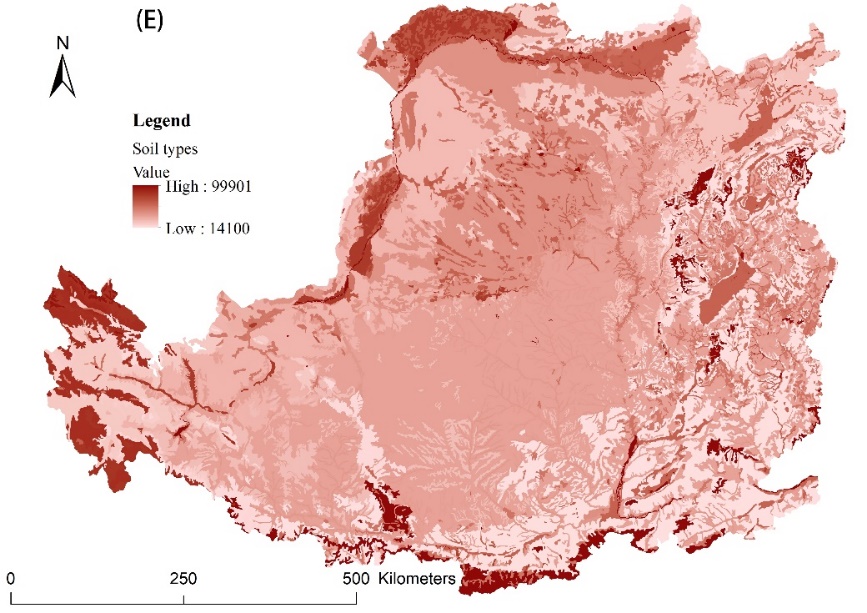


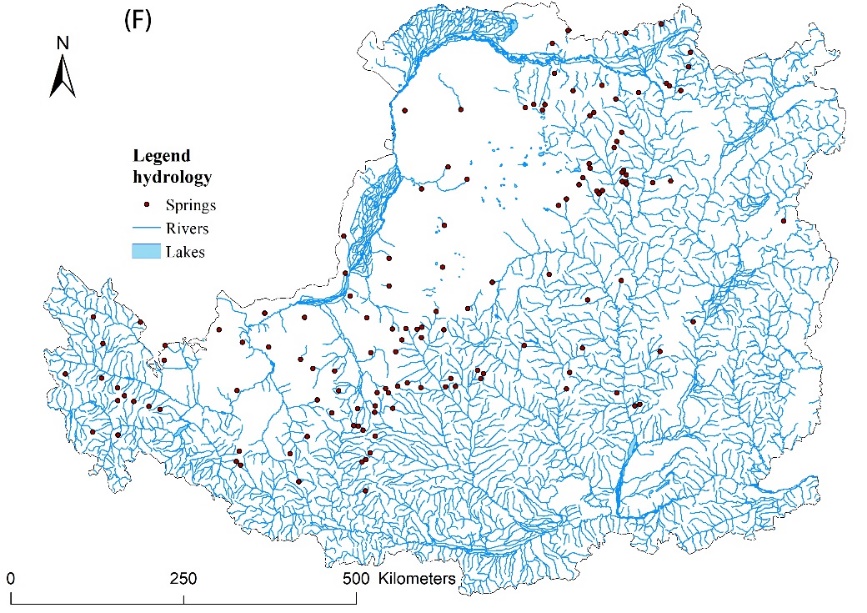


**Supplementary Fig. S4.** Distribution of components in terms of geological age (A), lithology (B), slope position (C), landform categories (D), soil types (E), and hydrology (F).

In order to evaluate the diversity of each element, a focal statistics analysis (neighborhood toolbox in ArcGIS 10.0 © ESRI) was applied to calculate diversity values [22]. The selected neighborhood was a circle with a 1km radius, and the diversity of each element was calculated accordingly. The radius was chosen considering the spatial unit for data at the coarsest resolution and also considering the ultimate aims of the *k*-means clustering. Geomorphological richness can be obtained by summing the diversity of slope positions and landforms. Soil richness included soil types from the 1:1 M scale China soil map. Hydrological elements were extracted from the 1:1 M national fundamental geographic map including rivers, lakes and springs. Terrain data were derived from the DEM as a ﬂoating raster
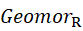
 or converted into a grid raster from a starting vector layer
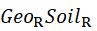
. Richness (referring to the variety) of each abiotic element can be calculated by geological, soil, geomorphological, and hydrological richness in the CLP, respectively (Supplementary Fig. S5). Total geo-richness (
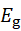
) can be obtained by the sum of the richness of the geological, geomorphological, soil and hydrological aspects (Supplementary Fig. S6), i.e.:


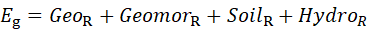


where
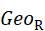
 is the classiﬁed raster map of geological age and lithology factor,
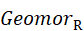
 is the classiﬁed raster map of the sum of slope position and landform category diversity factor,
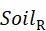
 is the classiﬁed raster map of the soil diversity factor and
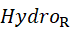
 is the classiﬁed raster map of the hydrological diversity factor.

The geodiversity index (GI) is calculated:


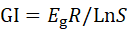


where *E*_g_ is the total geo-richness of different abiotic elements in the spatial unit; *R* is the coefficient of roughness of the spatial unit; *S* is the surface area of the spatial unit (m^2^); and Ln is the natural logarithm. This method considers roughness as the topographical aspect. The coefficient of roughness attempts to account for variation in orientation, slope and radiation, which plays an important role on associated energies and material fluxes, and in turn, the diversity and distribution of landforms, soils and processes [5]. The roughness coefficient was computed to a grid as the ratio between the real surface area and the planimetric one of the same square cell, akin to a landscape roughness in the DEM Surface Tools of ArcGIS [21]. The ratio has a wide range of uses, including habitat assessment, land use planning and geomorphology. The resultant geodiversity index is largely independent of scale [8, 22], and was used as one of 24 indicators for the regional classification of CZs in the CLP (Supplementary Fig. S7).


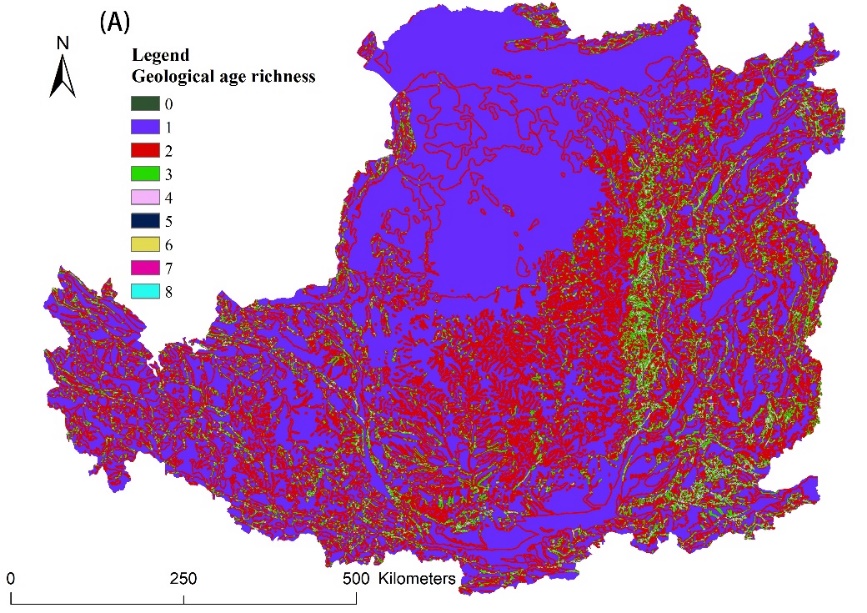


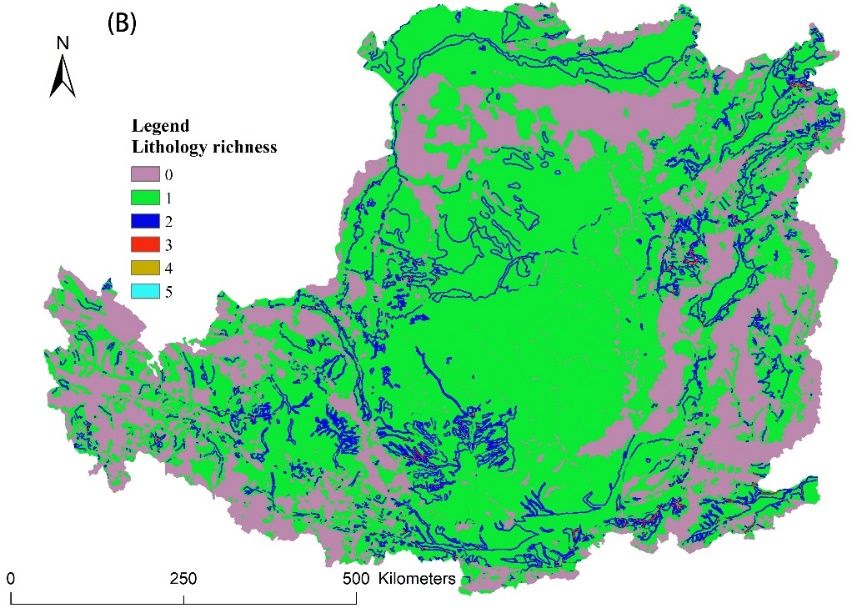


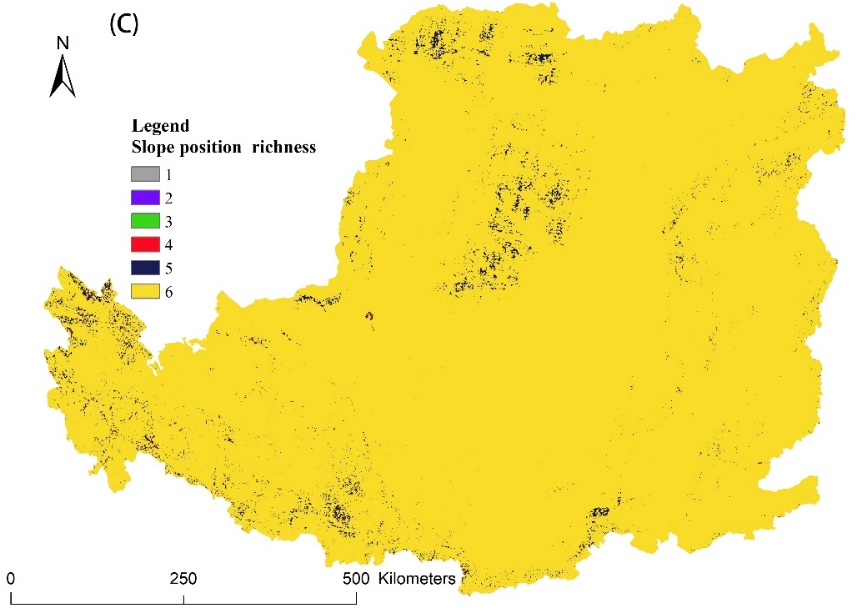


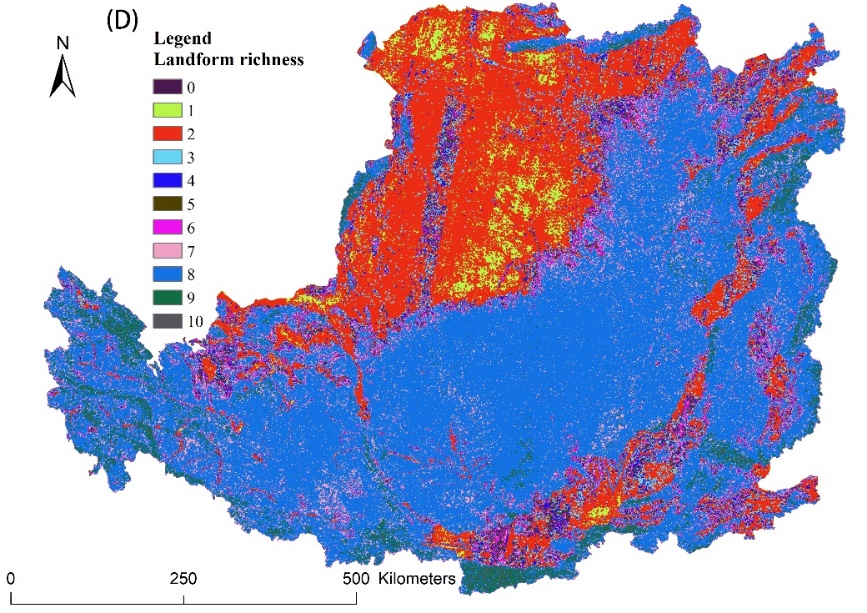


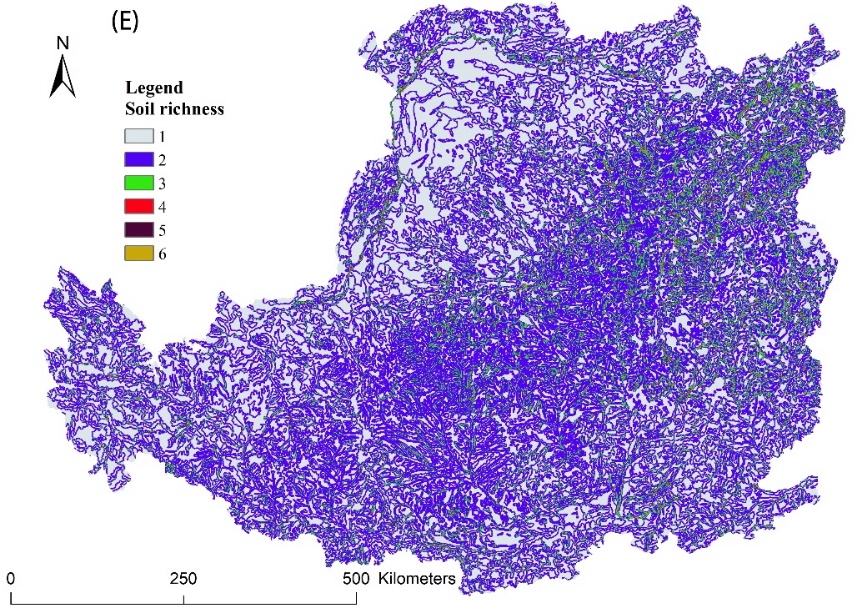


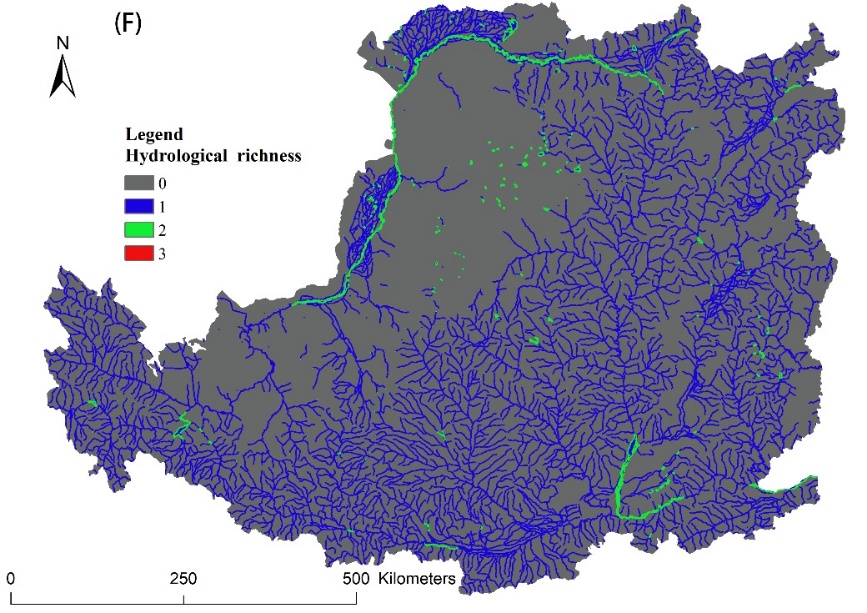


**Supplementary Fig. S5.** The richness of geological age (A), lithology (B), slope position (C), landform (D), soil types (E) and hydrology (F).

**
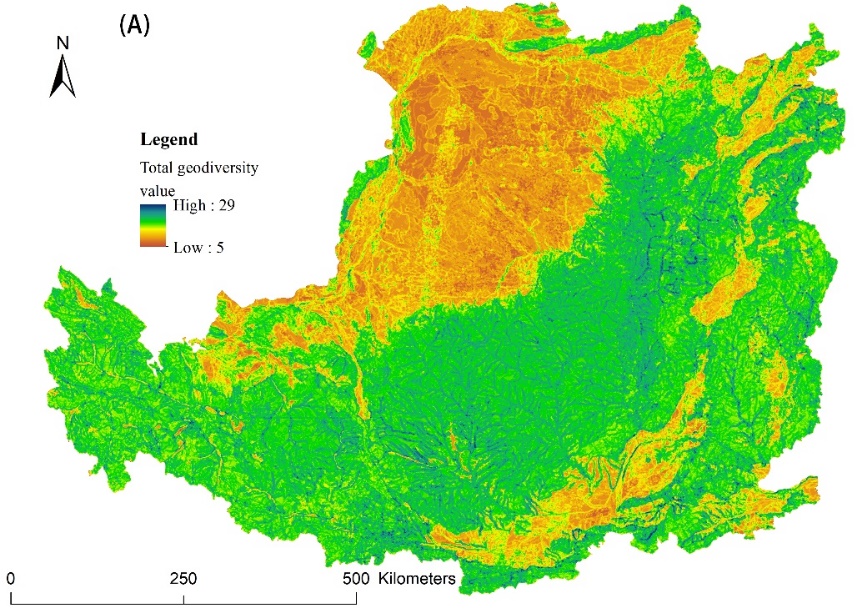

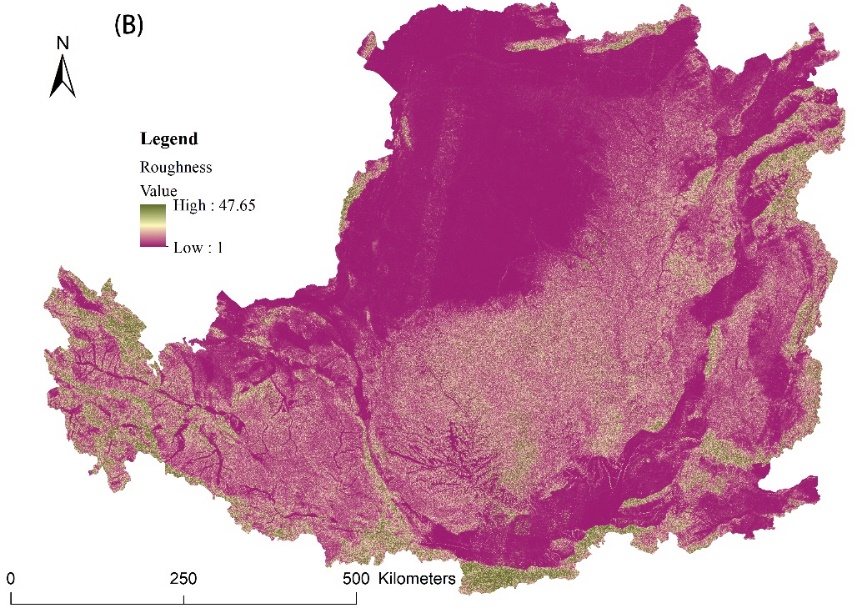
**

**Supplementary Fig. S6.** Total geodiversity (A) and roughness (B) of the CLP.

**
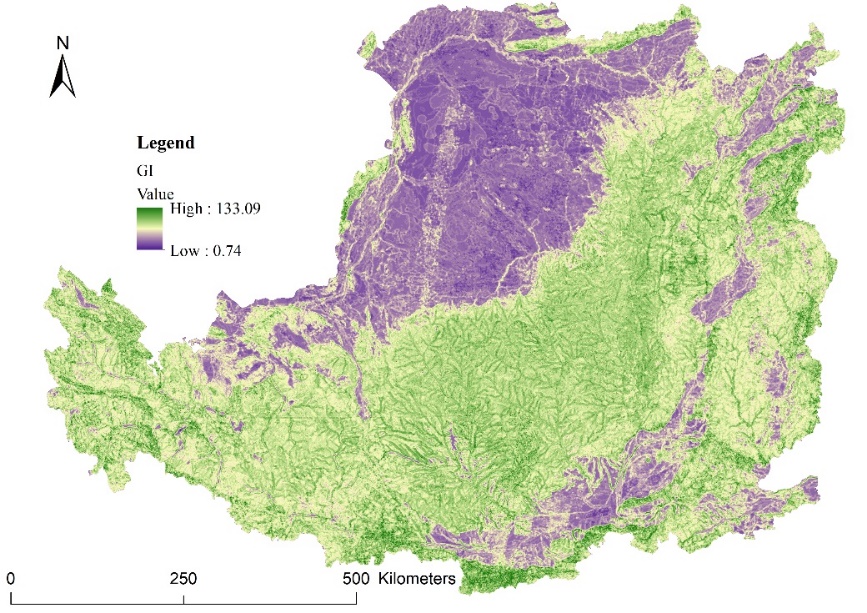
**

**Supplementary Fig. S7.** Geodiversity index (GI) of the CLP.

**Ecosystem, surface soil, and topography**

Ecosystems, the living and active upper layer of the CZ, should be considered as important components for characterizing CZs in the CLP. Here we chose the most relevant vegetation factors, such as the fraction of vegetation cover (FVC), net primary production (NPP) and the ratio of different vegetation types measured over regular hexagon spatial units, which were used to represent functional and structural indicators characterizing the regional variation of ecosystems. Annual mean FVC and NPP from 2000 to 2015 can be calculated from 250 m resolution datasets. Ratios of tree, shrub and grass cover to each 5 km width regular hexagon spatial unit (TREE, SHRUB and GRASS) were determined by using a 30m resolution land use map from 2010 [4, 25]. Soil properties are also essential for understanding the structure, evolution, and functions of CZs. Subsequently, we utilized soil properties data for the CLP based on a harmonized world soil database (HWSD). Database fields of soil organic content (OC), soil bulk density (SBD), soil particle size distribution and percentage of clay, silt, sand and gravel (CLAY, SILT, SAND and GRAVEL) were chosen for conversion to a 1 km resolution raster map. The compound topographic index (CTI) (ln (a/tan (b)), where a is the upslope contributing area and b is the slope degree of the landscape), represents the watershed scale surface wetness status that may influence the variations of CZ thickness as it closely relates to controls on eco-hydrological processes [26]. In this respect, CTI is also used as a key indicator in our classification framework and was calculated using the spatial analyst tools in ArcGIS 10.0. The spatial distributions of the 12 CZ indicators relevant to ecosystems, soils, and topography are shown in Supplementary Fig. S8.


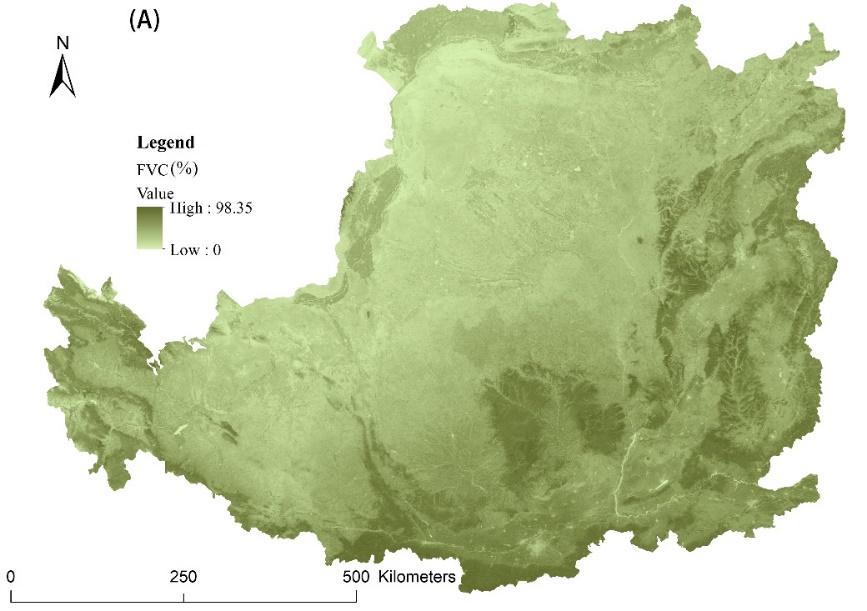


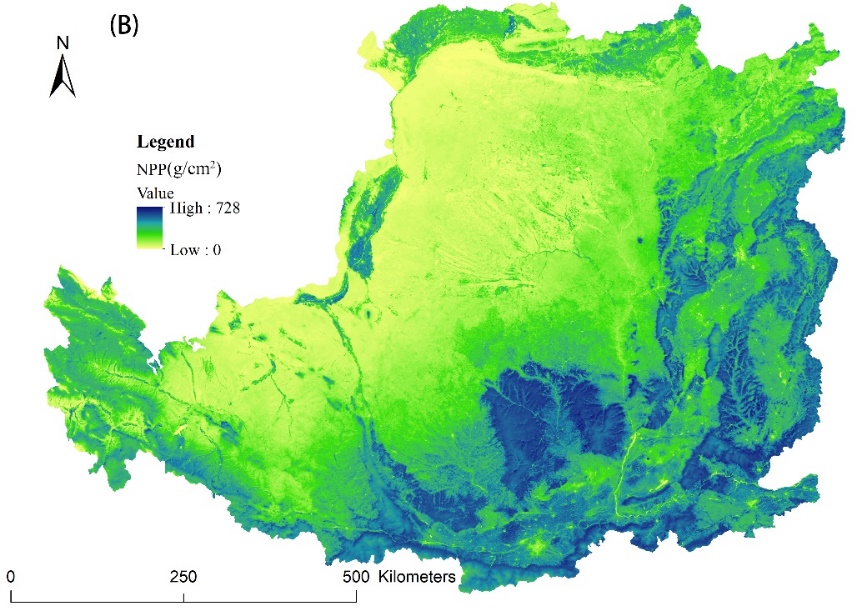


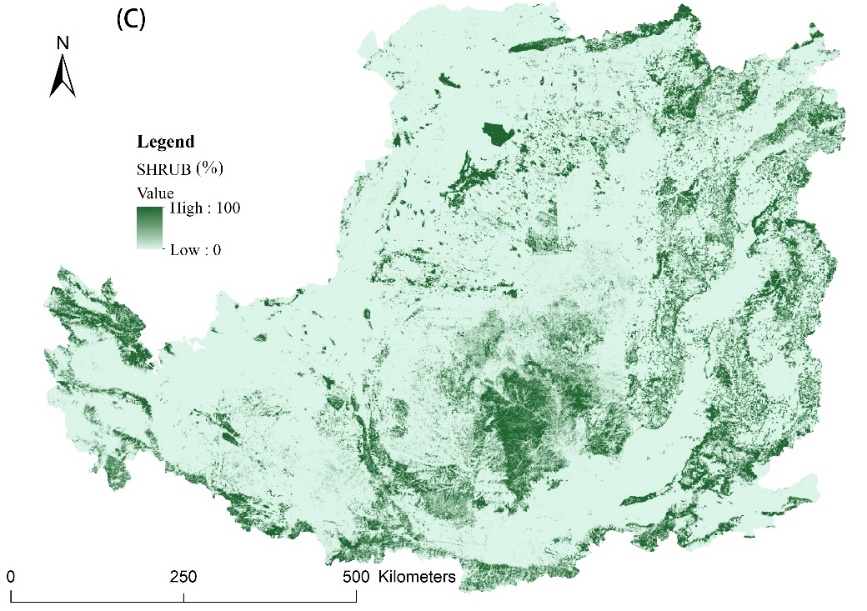

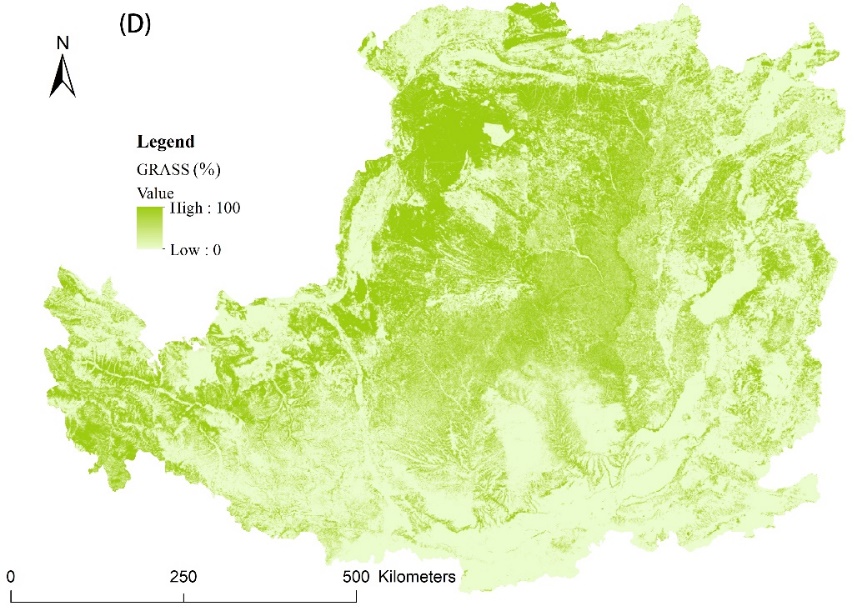

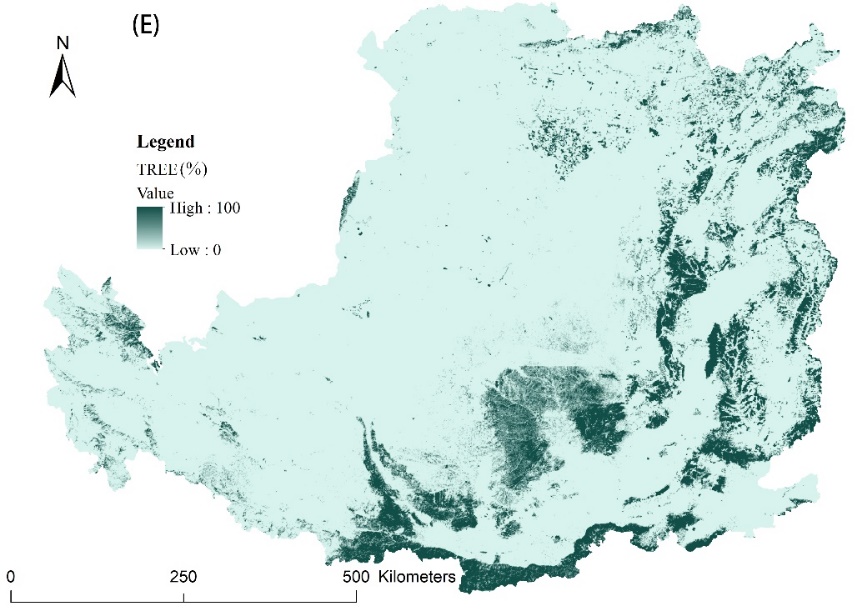

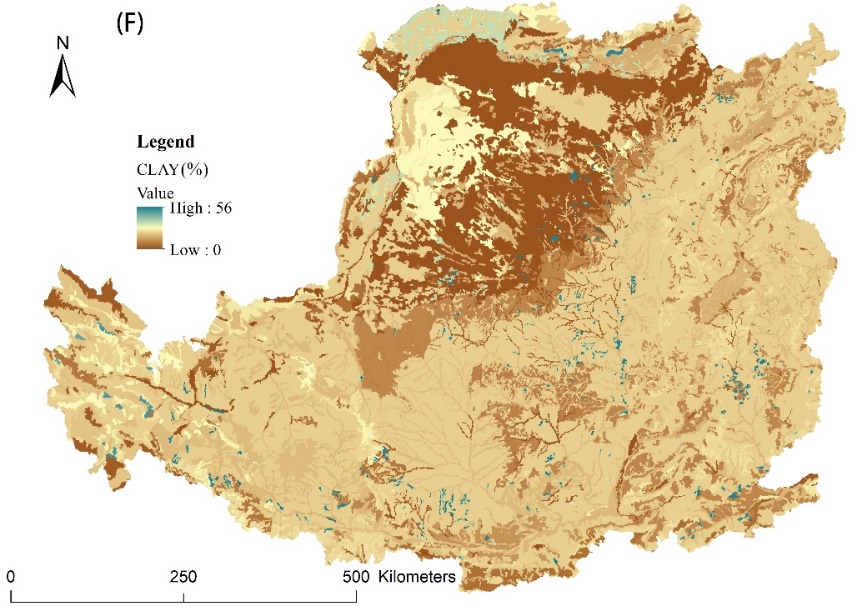

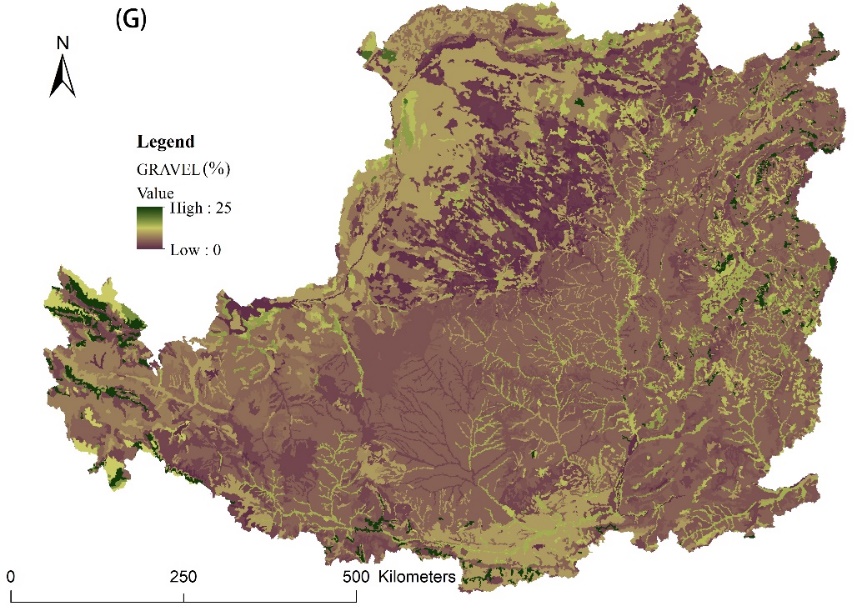

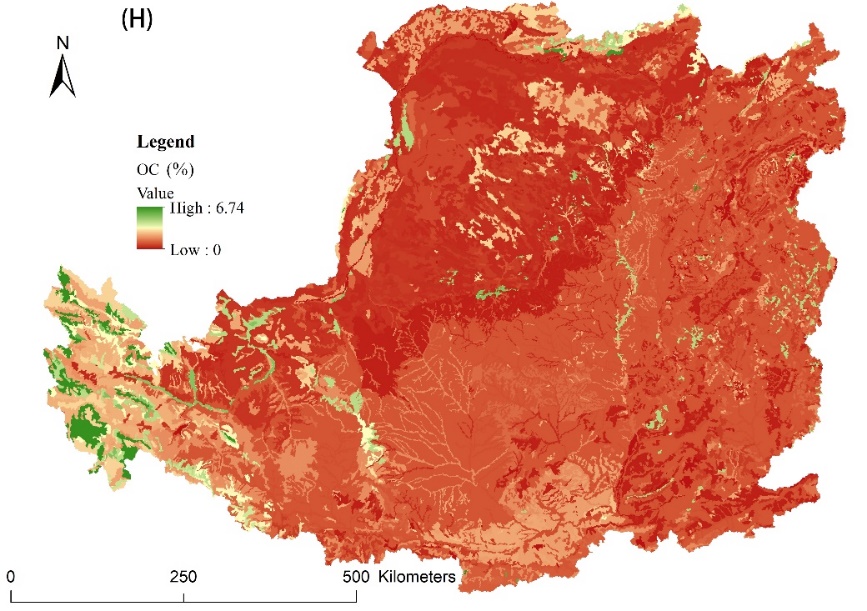

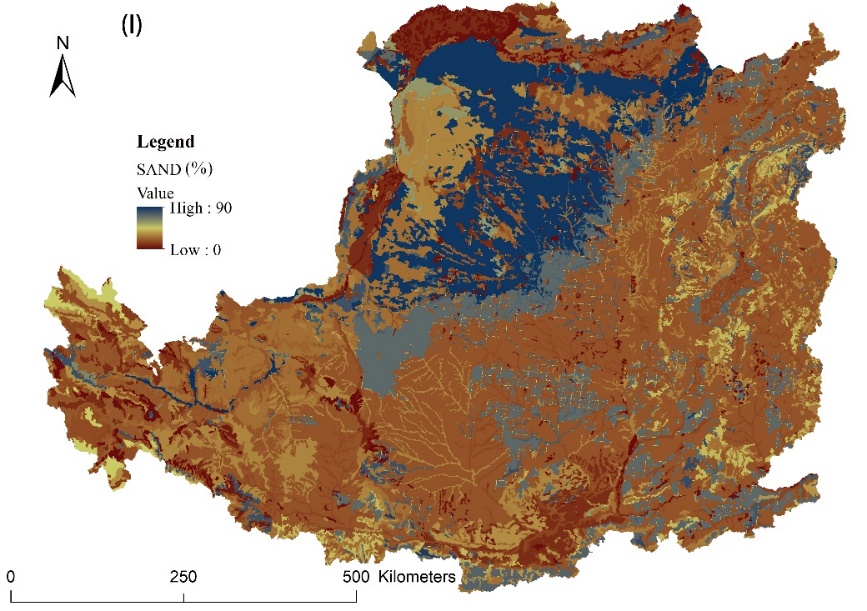

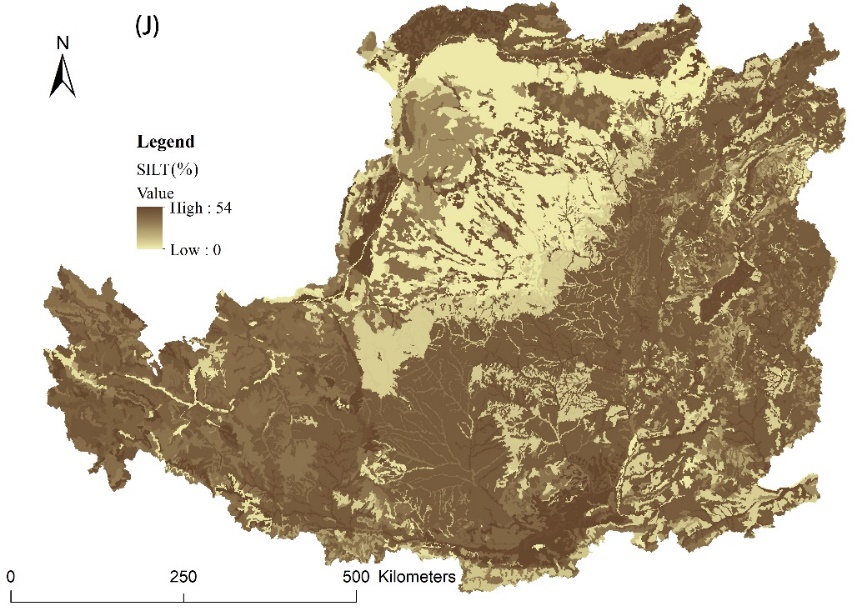


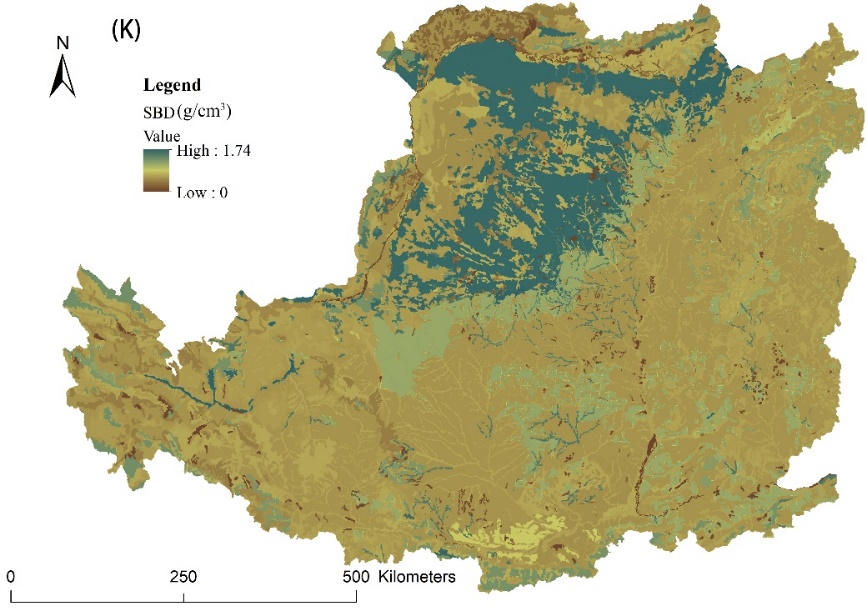

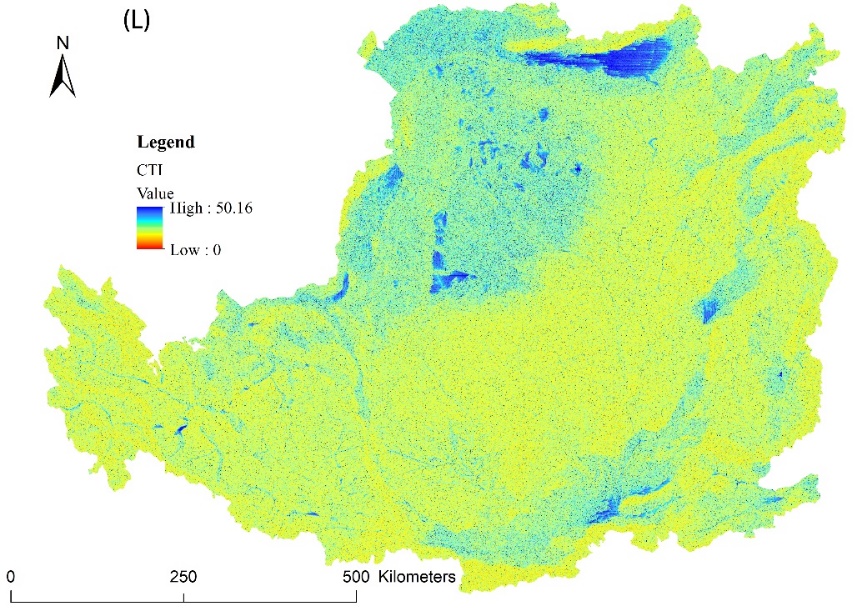


**Supplementary Fig. S8.** The CZ indicators from ecosystems, soils, and topography including fraction of vegetation cover (A), net primary production (B), ratios of shrub (C), ratios of grass (D), ratios of tree (E), clay (F), gravel (G), organic carbon (H), sand (I), silt (J), soil bulk density (K) and compound topographic index (L).

**Climate**

Climate operates as a driver modifying not only the Earth surface conditions but also the distributions of biota [19, 27]. For climate indicators, we spatially interpolated mean annual temperature (MAT) and mean annual precipitation (MAP) data from the 55 meteorological stations in the CLP using Anusplin software at a 1 km resolution [28]. Furthermore, we used the daily data of the same 55 meteorological stations to calculate potential evapotranspiration (PET) by the Penman-Monteith equation [29] and obtained the annual PET by the sum of the daily PET. The humidity index (HI) was then calculated as MAP/PET. We used the same interpolation method, as used for MAT and MAP, to obtain the spatial distribution of annual PET and HI at the 1 km resolution.

Effective energy and mass transfer (EEMT), consisting of heat energy and net primary production energy, is considered closely related to CZ structure and functions [26, 30]. Modeling or data-informed empirical equations are two popular methods to calculate EEMT [26, 30-35]. However, due to the absence of observed runoff data, we conducted a modeling method for EEMT (J m^-2^ s^-1^or W m^-2^) calculation [30, 33, 36], as follows:


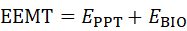
,

where
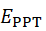
is heat energy related to effective precipitation energy and mass transfer and
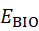
 is NPP energy and mass transfer:


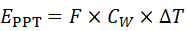
,

where *F* is available water mass ﬂux that is approximated using effective precipitation PPT − PET (kg m^−2^ s^−1^), PPT is monthly precipitation, PET is potential evapotranspiration calculated following Penman-Monteith equation to move into and through the subsurface (kg m^-2^s^-1^),
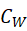
 is the speciﬁc heat of water (4.2×10^3^ J kg^-1^K^-1^), and
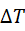
 = *T* _ambient_ -*T* _ref_ (K) with *T* _ambient_ as the ambient temperature in Fahrenheit at time of water ﬂux and *T* _ref_ being set at 273.15 K, and:


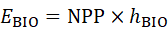
,


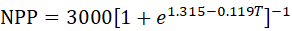
,

where
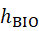
 is the speciﬁc enthalpy (J kg^-1^) at a ﬁxed value of 22 × 10^6^. Details of the three indicators can be found in Rasmussen and Gallo [33]. Calculated mean annual
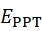
,
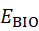
 and
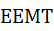
 were similarly interpolated at the 1 km resolution by Anusplin softwate across the CLP. All seven CZ indicators from climate data are shown in Supplementary Fig. S9.


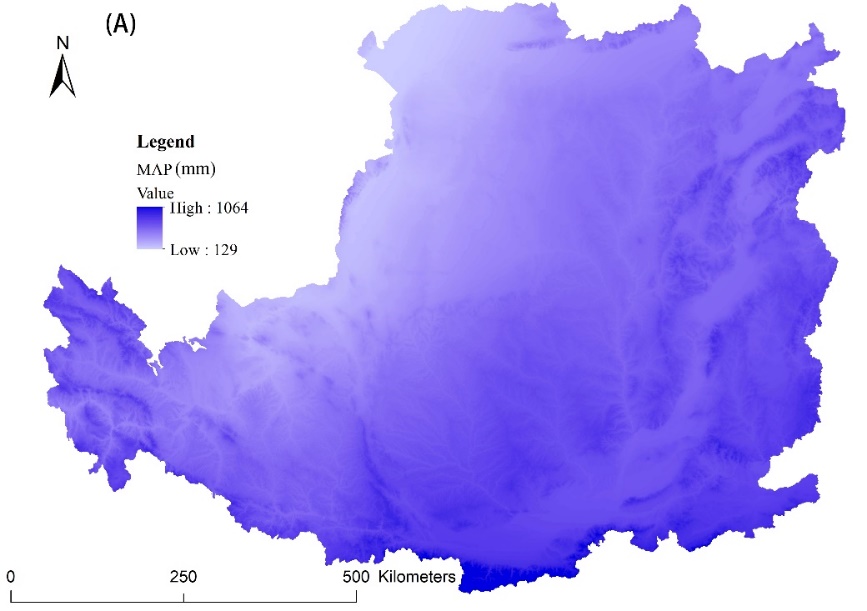

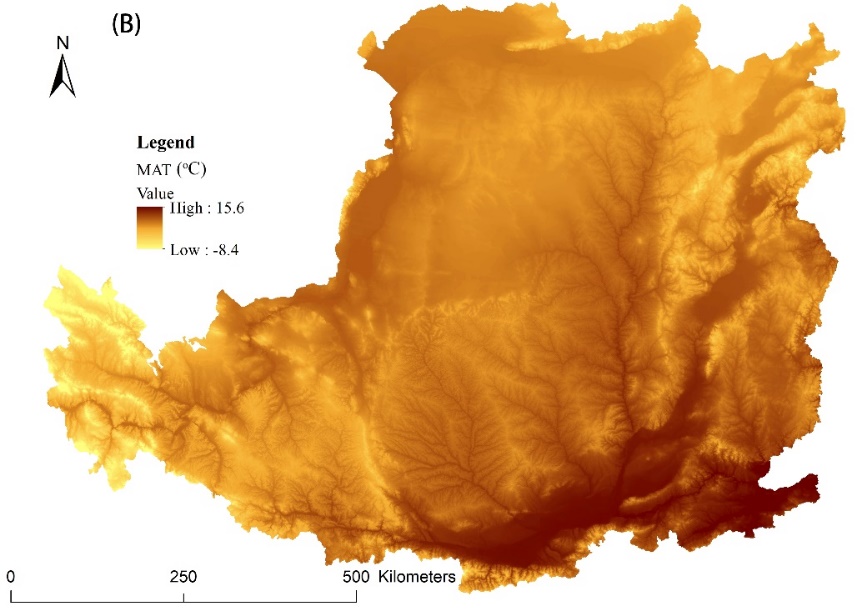

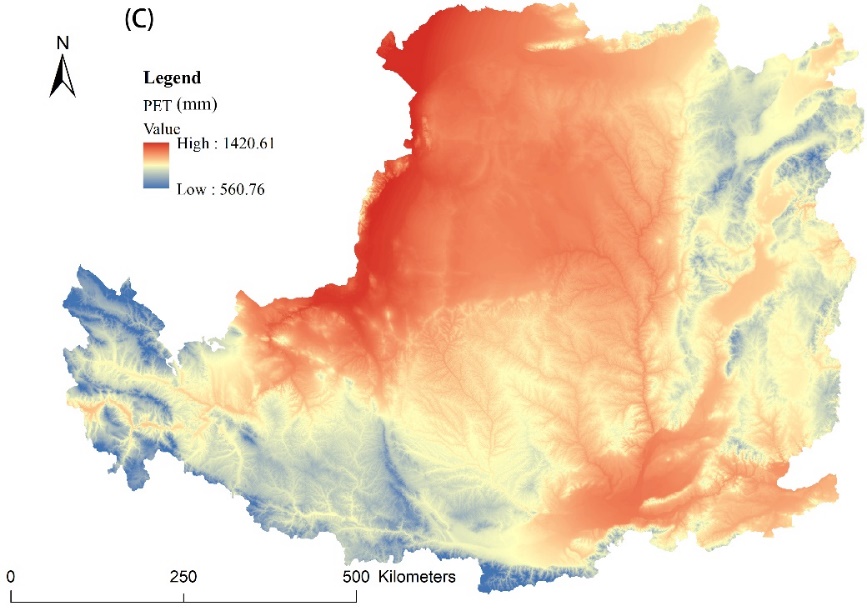

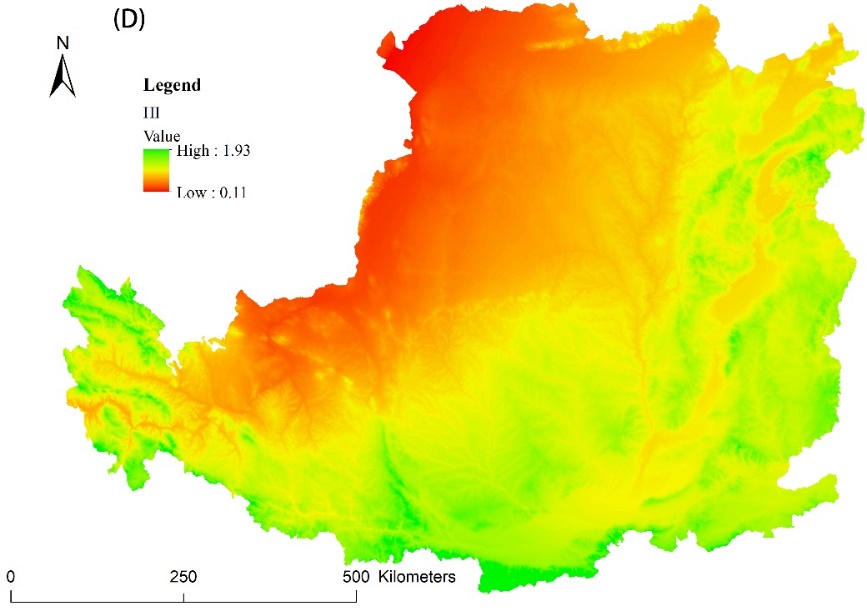


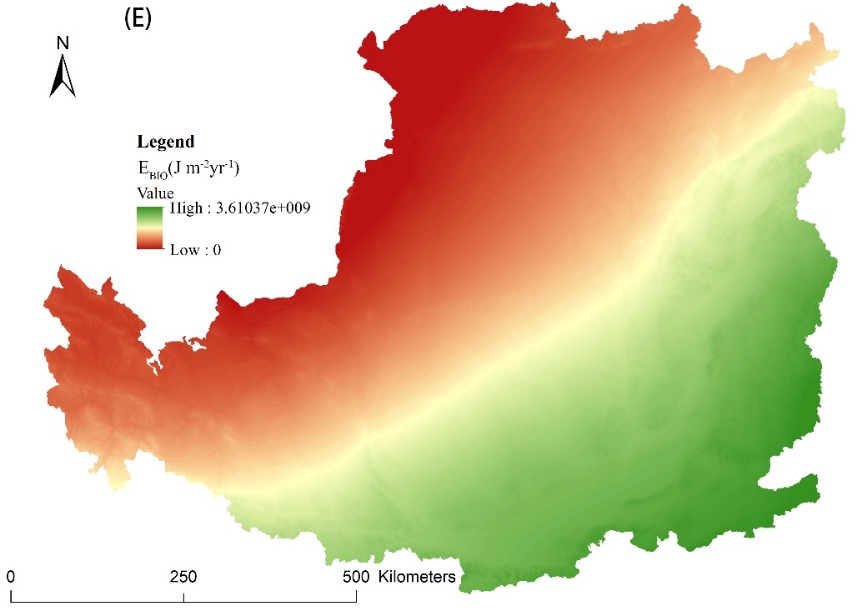

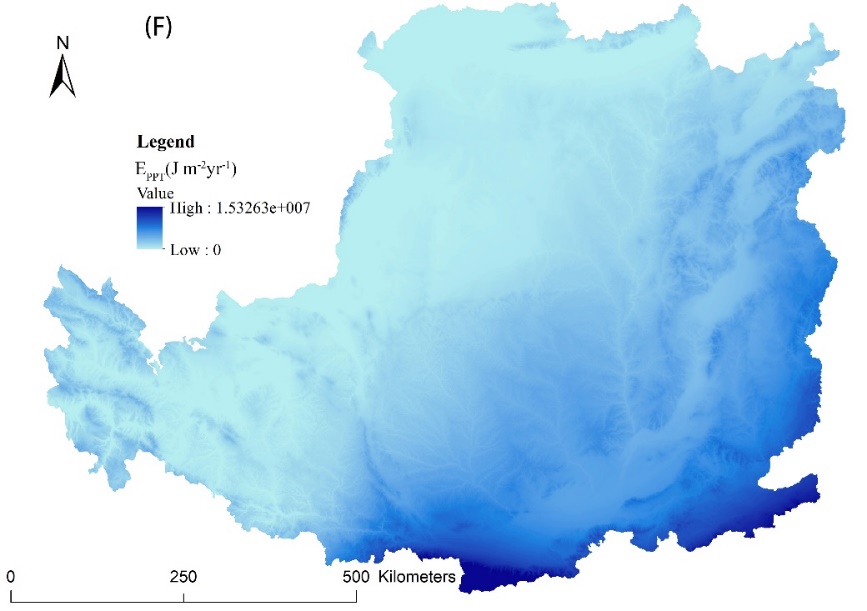


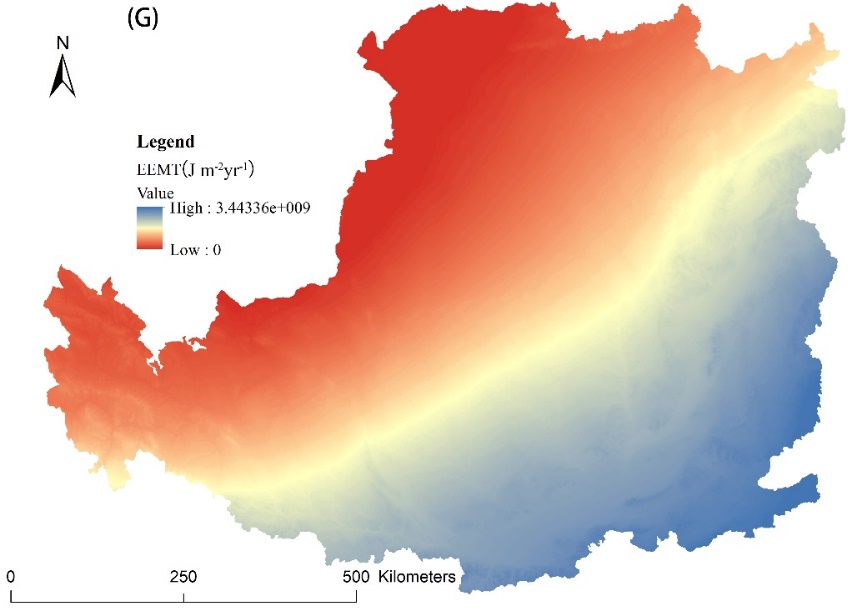


**Supplementary Fig. S9.** The CZ indicators from the climate including annual mean precipitation (A), annual mean temperature (B), potential evapotranspiration (C), humidity index (D), net primary production energy and mass transfer (E), heat energy related to effective precipitation energy and mass transfer (F) and effective energy and mass transfer (G).

**Human pressures and socio-economic development factors**

Humans have exerted huge impacts on CZs driven by the demands for food, materials, and living spaces. Hence, we also incorporated human and socioeconomic factors as driving forces for CZ change in the Anthropocene. Human-dominated land uses were extracted from a 30 m resolution land use map (Supplementary Table S4). Ratio (%) of artificial ground (AG) and farmland (CROP) were calculated to obtain their spatial distributions (Supplementary Fig. S10). These factors can be used as surrogates of human pressures to the CZs. We also added socioeconomic influence factors including gross domestic product (GDP) and population density (POP) in 2010 as indicators of socioeconomic impacts for the classification of regional CZs [37] (Supplementary Fig. S10).

**Supplementary Table S4. Pressure surrogates using human dominant land use types.**

| Types | Subtypes |
| --- | --- |
| Artificial ground | Urban area |
|  | Industrial area |
|  | Transportation land |
|  | Mining land |
| Farmland | Paddy field |
|  | Cropland |


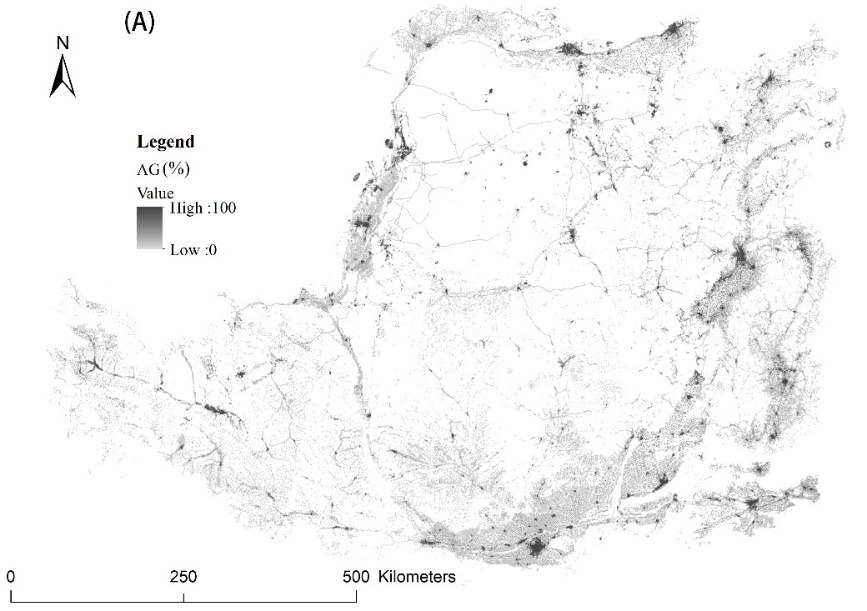

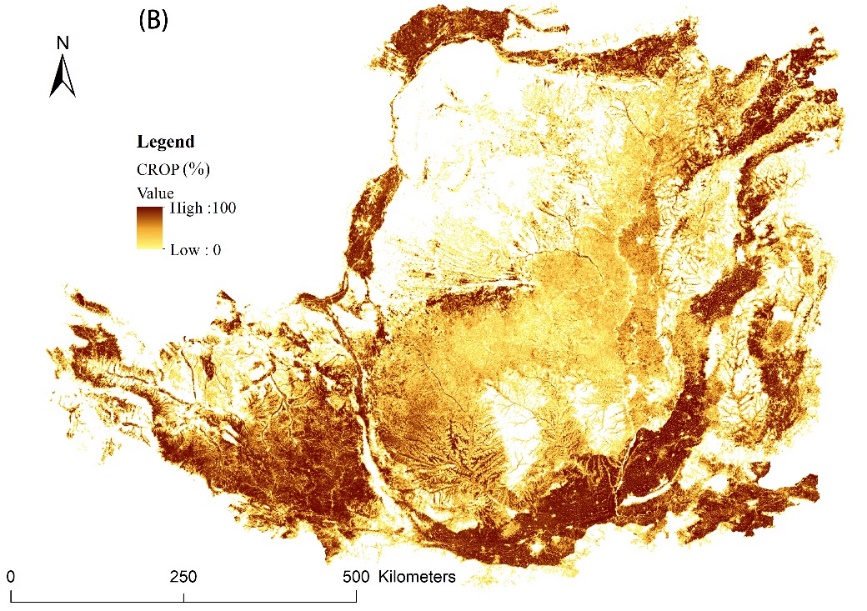

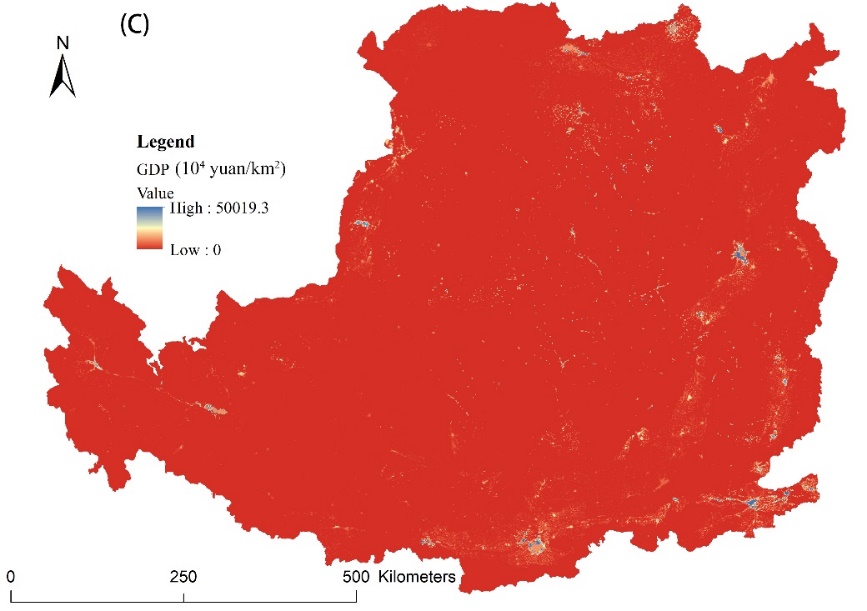


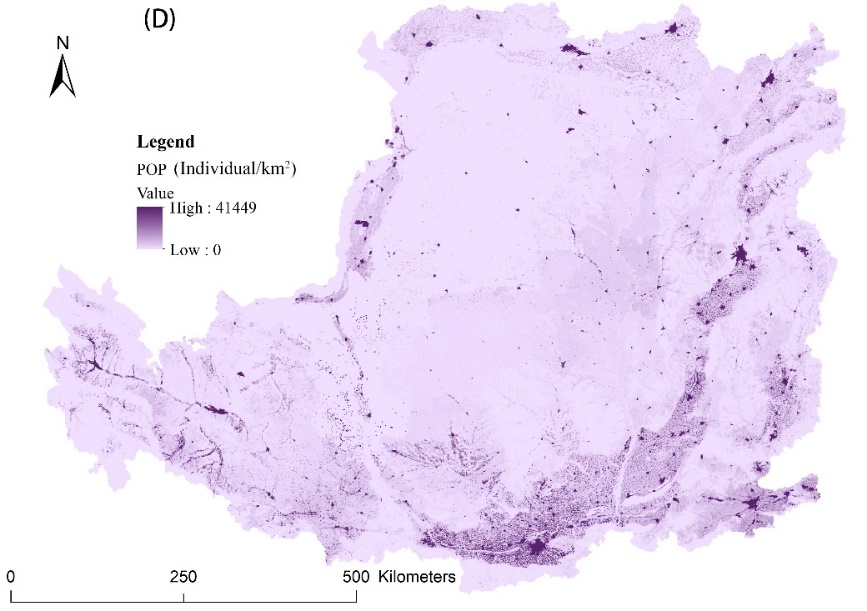


**Supplementary Fig. S10.** The CZ indicators from perspectives of human pressures and socioeconomic factors, including the ratio of artificial ground (A), the ratio of farmland (B), gross domestic product (C) and population density (D).

**Classification methods**

In this way, we considered geodiversity, ecosystems, climate, and anthropogenic indicators for classifying CZs in a regional context. Additionally, to overcome differences in scale and datasets at different spatial resolutions, all data were resampled to a single resolution of 1 km^2^ using a nearest neighbor approach (the NEAREST tool in ArcGIS 10.3), suitable for discriminating different CZ classes in the CLP. We chose a regular hexagon as the basic spatial unit for CZ classification of the CLP region because of its good performance in characterizing landscape heterogeneity and supporting spatial planning [38-40].

**Summary statistics and correlations of the CZ indicators**

In total, 24 CZ indicators were included in our classification framework (Fig. 1; Supplementary Table S5). Firstly, the mean values of the indicators were calculated for each spatial unit of a 5 km width regular hexagon using zonal statistics in an ArcGIS environment due to advanced computational efficiency and its correspondence to data at a 1 km resolution. Summary (descriptive) statistics and a correlation analysis were used to understand the basic characteristics and relationships for all 24 indicators (Supplementary Tables S5-6 and Fig. S11).

**Supplementary Table S5. Summary statistics of the 24 CZ indicators in the CLP.**

| **Components of CZ** | **CZ indicators** | **N** | **Min** | **Max** | | **Median** | | **Mean** | **Standard deviation** | **Coefficient of variation** |
| --- | --- | --- | --- | --- | --- | --- | --- | --- | --- | --- |
| A variety of CZ Physical components | GI | 29527 | 1.33 | 3.79 | | 2.66 | | 2.48 | 0.50 | 0.20 |
| Ecosystem | FVC (%) | 29527 | 0 | 77.44 | | 31.09 | | 34.13 | 16.79 | 0.49 |
|  | NPP (g/m^2^) | 29527 | 0 | 627.60 | | 249.64 | | 262.69 | 163.97 | 0.62 |
|  | TREE (%) | 29527 | 0 | 100.00 | | 0.01 | | 9.54 | 19.54 | 2.05 |
|  | SHRUB (%) | 29527 | 0 | 100.00 | | 3.48 | | 11.55 | 16.42 | 1.42 |
|  | GRASS (%) | 29527 | 0 | 100.00 | | 33.08 | | 37.24 | 29.57 | 0.79 |
|  | CLAY (%) | 29527 | 0 | 56.00 | | 20.03 | | 17.94 | 5.65 | 0.32 |
|  | GRAVEL (%) | 29527 | 0 | 25.00 | | 6.61 | | 7.41 | 3.07 | 0.41 |
|  | OC (%) | 29527 | 0 | 3.02 | | 0.65 | | 0.74 | 0.38 | 0.51 |
|  | SBD (g/cm^3^) | 29527 | 0 | 1.74 | | 1.41 | | 1.44 | 0.13 | 0.09 |
|  | SAND (%) | 29527 | 0 | 90.00 | | 41.14 | | 48.37 | 17.32 | 0.36 |
|  | SILT (%) | 29527 | 0 | 54.00 | | 38.00 | | 33.01 | 12.46 | 0.38 |
|  | CTI | 29527 | 0 | 32.41 | | 6.84 | | 7.25 | 1.54 | 0.21 |
| Climate | MAP (mm) | 29527 | 130.67 | 1057.58 | | 479.19 | | 464.38 | 136.24 | 0.29 |
|  | MAT (^o^C) | 29527 | -7.26 | 15.61 | | 8.42 | | 8.45 | 2.76 | 0.33 |
|  | PET (mm) | 29527 | 560.76 | 1420.61 | | 1135.14 | | 1124.94 | 112.99 | 0.10 |
|  | HI | 29527 | 0.11 | 1.93 | | 0.43 | | 0.41 | 0.15 | 0.37 |
|  | E_BIO_  (MJ m^-2^yr^-1^) | 29527 | 0 | 3720.00 | | 3720.00 | | 1610.00 | 1670.00 | 0.61 |
|  | E_PPT_  (MJ m^-2^yr^-1^) | 29527 | 0 | 15.60 | | 15.60 | | 1.90 | 2.55 | 0.81 |
|  | EEMT  (MJ m^-2^yr^-1^) | 29527 | 0 | 3570.00 | | 3570.00 | | 1520.00 | 1580.00 | 0.62 |
| Human and socioeconomic factors | AG (%) | 29527 | 0 | 99.72 | 0.28 | | 2.90 | | 7.40 | 2.56 |
|  | CROP (%) | 29527 | 0 | 99.73 | 21.86 | | 31.18 | | 30.21 | 0.97 |
|  | GDP  (10^4^ yuan/km^2^) | 29527 | 0 | 36457.34 | 32.12 | | 437.36 | | 1559.98 | 3.57 |
|  | POP  (Individual/km^2^) | 29527 | 0 | 25175.24 | 53.68 | | 172.67 | | 580.98 | 3.36 |

**Supplementary Table S6. Pearson correlation coefficients for the 24 CZ indicators in the CLP.**

|  | MAP | MAT | PET | HI | FVC | NPP | TREE | SHRUB | GRASS | CLAY | GRAVEL | OC | SBD | SAND | SILT | CTI | AG | CROP | GDP | POP | GI | E_BIO_ | E_PPT_ | EEMT |
| --- | --- | --- | --- | --- | --- | --- | --- | --- | --- | --- | --- | --- | --- | --- | --- | --- | --- | --- | --- | --- | --- | --- | --- | --- |
| MAP | 1.00 |  |  |  |  |  |  |  |  |  |  |  |  |  |  |  |  |  |  |  |  |  |  |  |
| MAT | 0.14 | 1.00 |  |  |  |  |  |  |  |  |  |  |  |  |  |  |  |  |  |  |  |  |  |  |
| PET | -0.67 | 0.47 | 1.00 |  |  |  |  |  |  |  |  |  |  |  |  |  |  |  |  |  |  |  |  |  |
| HI | 0.96 | -0.05 | -0.77 | 1.00 |  |  |  |  |  |  |  |  |  |  |  |  |  |  |  |  |  |  |  |  |
| FVC | 0.85 | 0.17 | -0.55 | 0.82 | 1.00 |  |  |  |  |  |  |  |  |  |  |  |  |  |  |  |  |  |  |  |
| NPP | 0.77 | 0.25 | -0.45 | 0.72 | 0.95 | 1.00 |  |  |  |  |  |  |  |  |  |  |  |  |  |  |  |  |  |  |
| TREE | 0.49 | -0.06 | -0.36 | 0.51 | 0.65 | 0.58 | 1.00 |  |  |  |  |  |  |  |  |  |  |  |  |  |  |  |  |  |
| SHRUB | 0.35 | -0.20 | -0.32 | 0.37 | 0.44 | 0.43 | 0.35 | 1.00 |  |  |  |  |  |  |  |  |  |  |  |  |  |  |  |  |
| GRASS | -0.39 | -0.23 | 0.26 | -0.38 | -0.46 | -0.53 | -0.38 | -0.26 | 1.00 |  |  |  |  |  |  |  |  |  |  |  |  |  |  |  |
| CLAY | 0.15 | 0.03 | -0.17 | 0.16 | 0.25 | 0.29 | 0.08 | 0.04 | -0.09 | 1.00 |  |  |  |  |  |  |  |  |  |  |  |  |  |  |
| GRAVEL | 0.04 | -0.13 | -0.11 | 0.10 | 0.10 | 0.09 | 0.06 | 0.07 | -0.04 | 0.17 | 1.00 |  |  |  |  |  |  |  |  |  |  |  |  |  |
| OC | 0.13 | -0.42 | -0.36 | 0.23 | 0.18 | 0.14 | 0.02 | 0.15 | -0.02 | 0.32 | 0.28 | 1.00 |  |  |  |  |  |  |  |  |  |  |  |  |
| SBD | -0.13 | -0.07 | 0.20 | -0.12 | -0.20 | -0.25 | -0.03 | **0.00** | 0.15 | -0.55 | -0.04 | -0.14 | 1.00 |  |  |  |  |  |  |  |  |  |  |  |
| SAND | -0.21 | -0.05 | 0.26 | -0.21 | -0.27 | -0.32 | -0.02 | -0.02 | 0.18 | -0.82 | -0.17 | -0.36 | 0.82 | 1.00 |  |  |  |  |  |  |  |  |  |  |
| SILT | 0.25 | 0.03 | -0.26 | 0.26 | 0.29 | 0.33 | **0.01** | 0.03 | -0.18 | 0.78 | 0.22 | 0.42 | -0.55 | -0.92 | 1.00 |  |  |  |  |  |  |  |  |  |
| CTI | -0.49 | 0.18 | 0.55 | -0.51 | -0.42 | -0.34 | -0.32 | -0.30 | **0.00** | -0.23 | -0.07 | -0.19 | 0.14 | 0.19 | -0.20 | 1.00 |  |  |  |  |  |  |  |  |
| AG | **0.00** | 0.26 | 0.12 | -0.04 | -0.03 | 0.02 | -0.13 | -0.17 | -0.24 | 0.02 | 0.06 | **-0.01** | -0.10 | -0.09 | 0.08 | 0.21 | 1.00 |  |  |  |  |  |  |  |
| CROP | 0.12 | 0.37 | **0.00** | 0.07 | 0.04 | 0.16 | -0.32 | -0.36 | -0.48 | 0.19 | -0.07 | -0.04 | -0.19 | -0.29 | 0.31 | 0.19 | 0.22 | 1.00 |  |  |  |  |  |  |
| GDP | 0.03 | 0.23 | 0.09 | **-0.01** | **-0.01** | 0.02 | -0.09 | -0.12 | -0.17 | **0.00** | 0.04 | **-0.01** | -0.06 | -0.05 | 0.04 | 0.15 | 0.76 | 0.14 | 1.00 |  |  |  |  |  |
| POP | 0.09 | 0.24 | 0.03 | 0.05 | 0.03 | 0.05 | -0.10 | -0.13 | -0.20 | 0.03 | 0.02 | **0.00** | -0.13 | -0.11 | 0.08 | 0.10 | 0.74 | 0.20 | 0.73 | 1.00 |  |  |  |  |
| GI | 0.62 | -0.11 | -0.58 | 0.64 | 0.56 | 0.49 | 0.35 | 0.32 | -0.11 | 0.31 | 0.07 | 0.21 | -0.23 | -0.33 | 0.35 | -0.69 | -0.19 | -0.04 | -0.14 | -0.08 | 1.00 |  |  |  |
| E_BIO_ | 0.88 | 0.50 | -0.37 | 0.77 | 0.79 | 0.79 | 0.40 | 0.23 | -0.44 | 0.19 | **-0.01** | -0.09 | -0.17 | -0.23 | 0.25 | -0.35 | 0.11 | 0.26 | 0.11 | 0.16 | 0.48 | 1.00 |  |  |
| E_PPT_ | 0.84 | 0.42 | -0.37 | 0.78 | 0.78 | 0.74 | 0.51 | 0.27 | -0.42 | 0.13 | 0.09 | -0.03 | -0.12 | -0.14 | 0.15 | -0.32 | 0.08 | 0.10 | 0.11 | 0.13 | 0.40 | 0.87 | 1.00 |  |
| EEMT | 0.88 | 0.50 | -0.38 | 0.78 | 0.79 | 0.79 | 0.40 | 0.23 | -0.45 | 0.20 | **-0.01** | -0.08 | -0.17 | -0.24 | 0.26 | -0.35 | 0.11 | 0.27 | 0.11 | 0.16 | 0.48 | 1.00 | 0.87 | 1.00 |

Note: the format of bold refers to no significant correlation at the level of *p*=0.05 while others represent significant correlation at the level of *p*=0.01.

**The classification of regional CZs in the CLP through PCA and clustering**

Because of the overwhelming complexity of the CZs at the regional scale, we chose not to give weightings to the 24 different indicators but determined their salience to the classification using a PCA process. This avoided introducing subjectivity to the classification process. Due to strong correlations between some CZ indicators and to reduce data redundancy (Supplementary Table S6), a PCA was applied. The PC scores of the first six PCs were used as inputs to a *k*-means clustering algorithm to derive homogeneous CZ classes [41, 42] (Supplementary Tables S7-8 and Fig. S11). The retained PCs were those with eigenvalues greater than 1. The raw indicator data were re-scaled by their mean and standard deviation to ensure that the CZ indicator measurements were comparable before input into the PCA. The re-scaling formula was as follows:

Z_Score=[x-mean]/sd

where Z_Score is standardized score; x is the raw indicator data; sd is standard deviation of the raw indicator data.

**
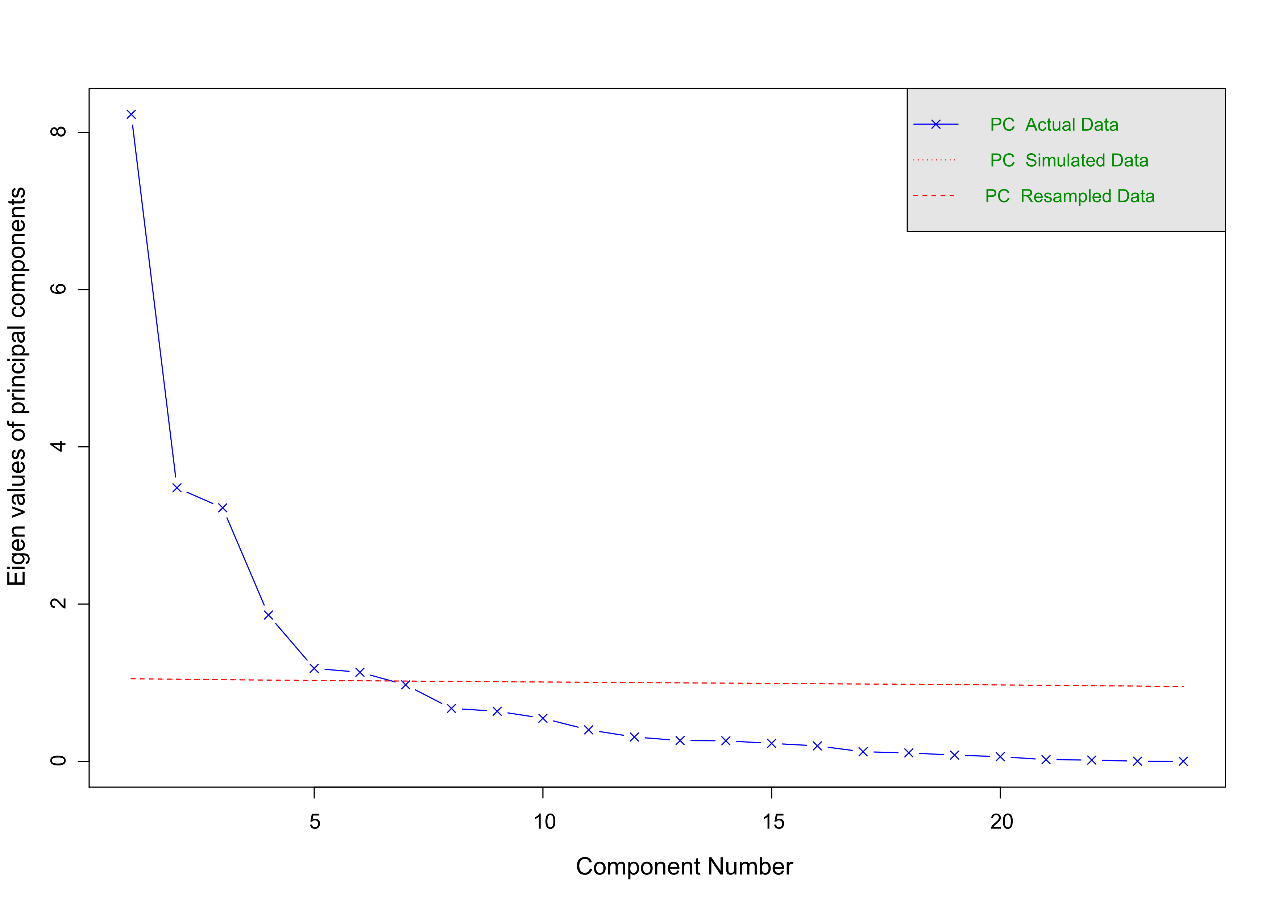
**

**Supplementary Fig. S11.** PCA scree plot with complementary analyses.

**Supplementary Table S7. The contribution of variances explained by the first six principal components.**

|  | Comp.1 | Comp.2 | Comp.3 | Comp.4 | Comp.5 | Comp.6 |
| --- | --- | --- | --- | --- | --- | --- |
| Standard deviation | 2.8688 | 1.8652 | 1.7956 | 1.3638 | 1.0872 | 1.0643 |
| Proportion of Variance | 0.3429 | 0.1450 | 0.1343 | 0.0775 | 0.0493 | 0.0472 |
| Cumulative Proportion | 0.3429 | 0.4879 | 0.6222 | 0.6997 | 0.7490 | 0.7962 |

Note: Comp. n represent the n^th^ principal component.

**Supplementary Table S8. PCA loadings of the CZ indicators for the first six components.**

| ECZ's indicators | Comp.1 | Comp.2 | Comp.3 | Comp.4 | Comp.5 | Comp.6 |
| --- | --- | --- | --- | --- | --- | --- |
| MAP | -0.323 | -0.042 | 0.113 | -0.034 | 0.152 | -0.093 |
| MAT | -0.062 | 0.335 | 0.221 | 0.353 | -0.020 | 0.237 |
| PET | 0.226 | 0.194 | 0.109 | 0.240 | -0.195 | 0.317 |
| HI | -0.314 | -0.100 | 0.066 | -0.111 | 0.129 | -0.163 |
| FVC | -0.320 | -0.051 | 0.076 | -0.004 | -0.154 | 0.042 |
| NPP | -0.309 | 0.015 | 0.064 | 0.051 | -0.223 | 0.016 |
| TREE | -0.198 | -0.174 | 0.121 | -0.075 | -0.326 | 0.218 |
| SHRUB | -0.142 | -0.220 | 0.036 | -0.157 | -0.341 | 0.174 |
| GRASS | 0.178 | -0.167 | -0.090 | 0.014 | 0.409 | 0.380 |
| CLAY | -0.135 | 0.113 | -0.402 | 0.130 | -0.062 | 0.191 |
| GRAVEL | -0.042 | -0.015 | -0.144 | -0.213 | -0.353 | 0.012 |
| OC | -0.071 | -0.087 | -0.311 | -0.295 | -0.116 | -0.243 |
| SBD | 0.116 | -0.161 | 0.332 | -0.119 | -0.008 | -0.199 |
| SAND | 0.155 | -0.172 | 0.442 | -0.116 | 0.038 | -0.111 |
| SILT | -0.158 | 0.149 | -0.415 | 0.088 | -0.041 | 0.009 |
| CTI | 0.187 | 0.221 | 0.089 | 0.081 | -0.389 | -0.170 |
| AG | -0.009 | 0.398 | 0.056 | -0.383 | 0.023 | 0.074 |
| CROP | -0.056 | 0.326 | -0.046 | 0.242 | 0.079 | -0.597 |
| GDP | -0.014 | 0.361 | 0.077 | -0.422 | 0.084 | 0.143 |
| POP | -0.034 | 0.367 | 0.056 | -0.396 | 0.147 | 0.101 |
| GI | -0.237 | -0.166 | -0.109 | -0.014 | 0.328 | 0.078 |
| E_BIO_ | -0.304 | 0.105 | 0.173 | 0.138 | 0.100 | 0.039 |
| E_PPT_ | -0.289 | 0.053 | 0.196 | 0.049 | -0.023 | 0.089 |
| EEMT | -0.306 | 0.106 | 0.170 | 0.135 | 0.098 | 0.029 |

Note: Comp. n represents for the n principal component.

The within-group sum of squares statistic (the total within-groups sums of squares against the number of clusters in a *k*-means solution) and a pseudo F-Statistic (a ratio reflecting within-group similarity and between-group differences) were used to determine the optimal number of clusters or classes (Supplementary Figs. S12-13). ArcGIS 10.3 software was used to prepare and calculate the critical indicators and display the spatial distributions of the CZ classes. R 3.4.1 software was used to conduct the summary statistics, correlation analysis, PCA and *k*-means clustering [43].

**
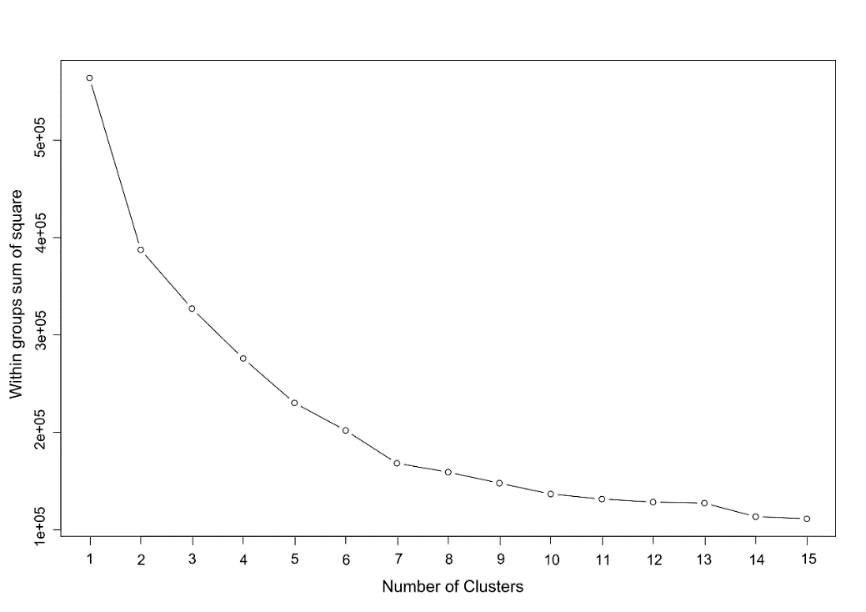
**

**Supplementary Fig. S12.** Within-group sum of squares with the *k*-means cluster analysis.

**
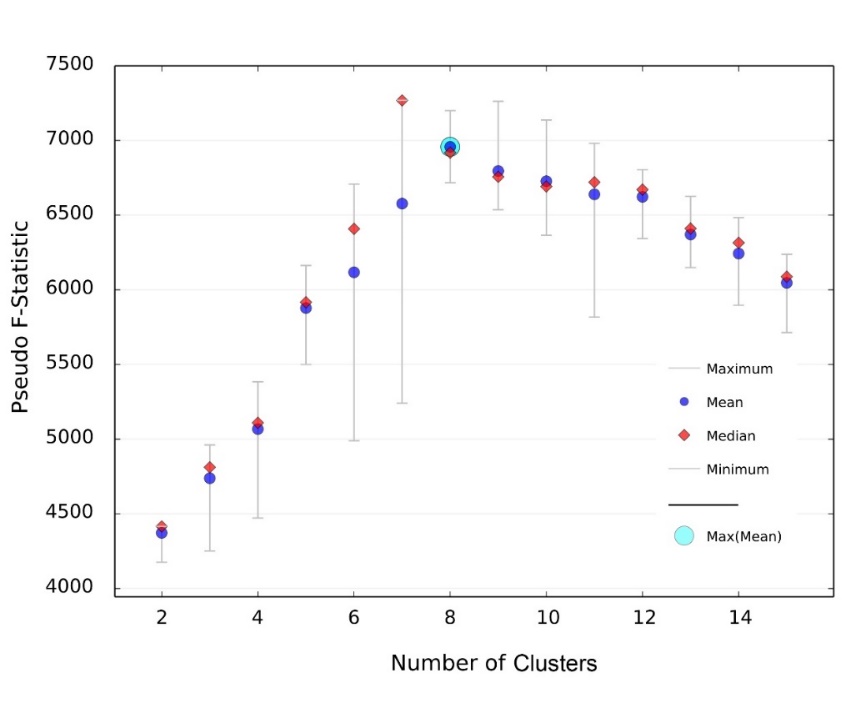
**

**Supplementary Fig. S13.** Pseudo F-statistic from the *k*-means cluster analysis.

**Geodiversity, ecosystem, surface soil, topography, climate, and natural and anthropogenic characteristics for each of the eight regional CZ classes**

We used generalization and areal tools in ArcGIS to dissolve similar classes and calculate area and percentage of each CZ class in the CLP region (Supplementary Fig. S14). The CZ classes were characterized by error-bar plots (Supplementary Fig. S15-20).


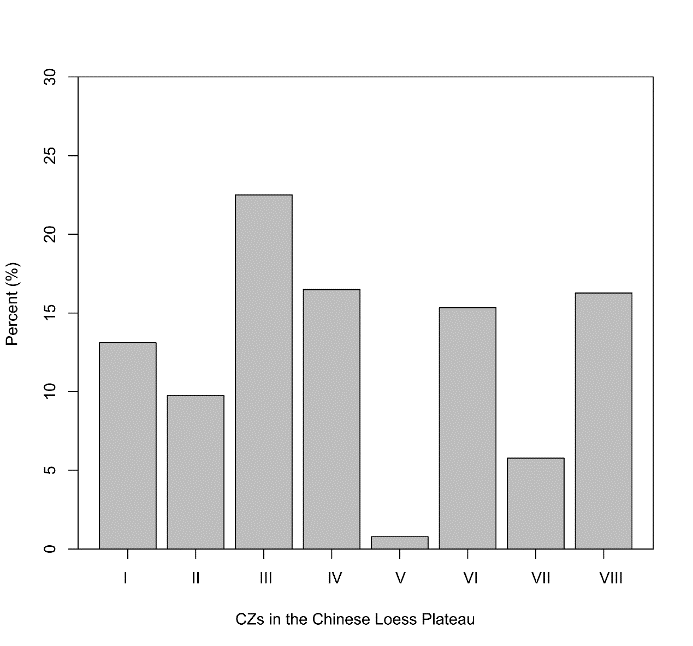


**Supplementary Fig. S14.** The percentage area of each CZ class in the CLP. The sample sizes (n) of Classes I, II, III, IV, V, VI, VII and VIII are 3870, 2878, 6648, 4868, 229, 4526, 1704 and 4804, respectively.


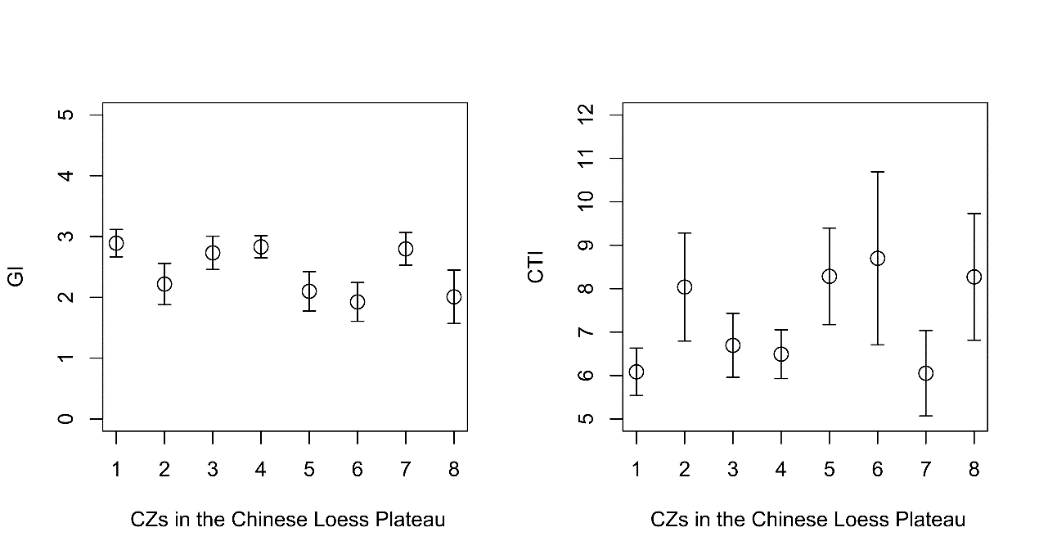


**Supplementary Fig. S15.** Geodiversity index (GI) and compound topographic index (CTI) for the eight CZ types in the CLP. The sample size (n) of Classes I, II, III, IV, V, VI, VII and VIII are 3870, 2878, 6648, 4868, 229, 4526, 1704 and 4804, respectively.


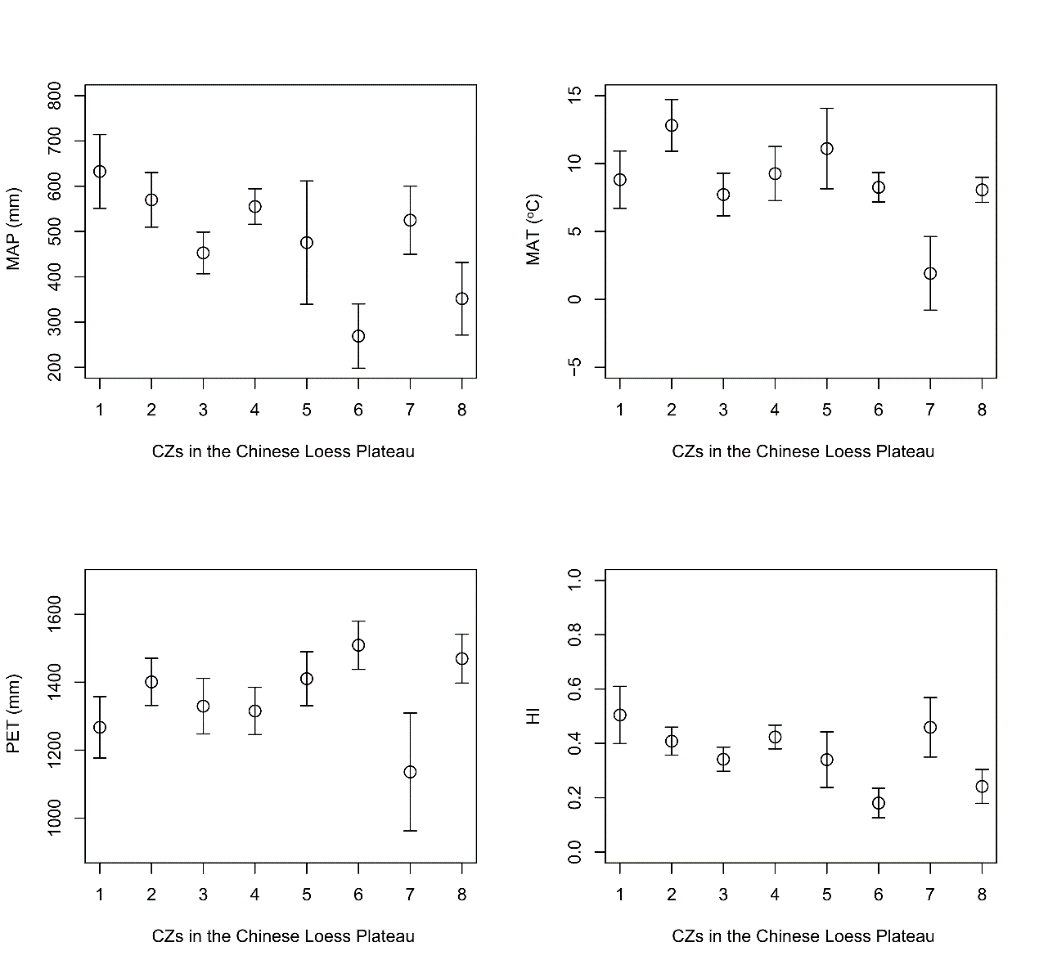


**Supplementary Fig. S16.** Climate indicators for the eight CZ classes in the CLP. The sample size (n) of Classes I, II, III, IV, V, VI, VII and VIII are 3870, 2878, 6648, 4868, 229, 4526, 1704 and 4804, respectively.


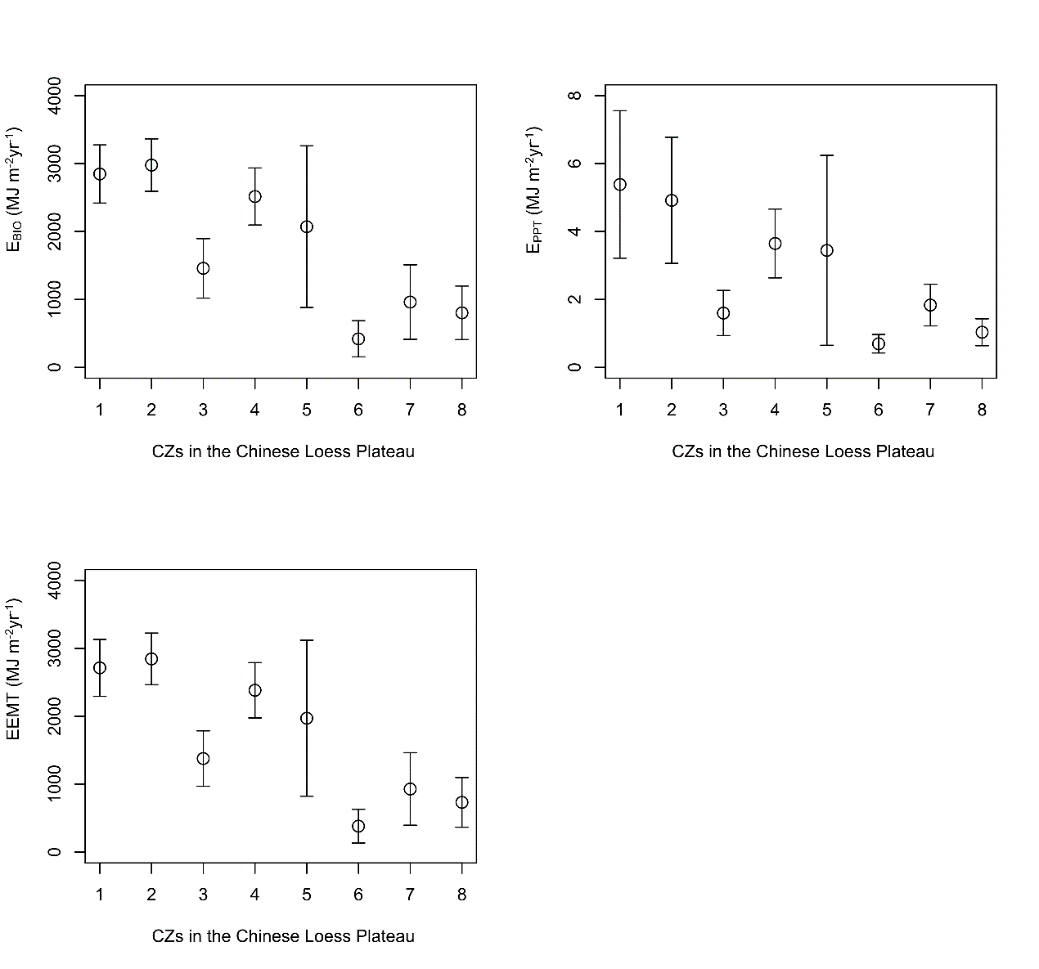


**Supplementary Fig. S17.** Effective energy and mass transfer indicators for the eight CZ classes in the CLP. The sample size (n) of Classes I, II, III, IV, V, VI, VII and VIII are 3870, 2878, 6648, 4868, 229, 4526, 1704 and 4804, respectively.


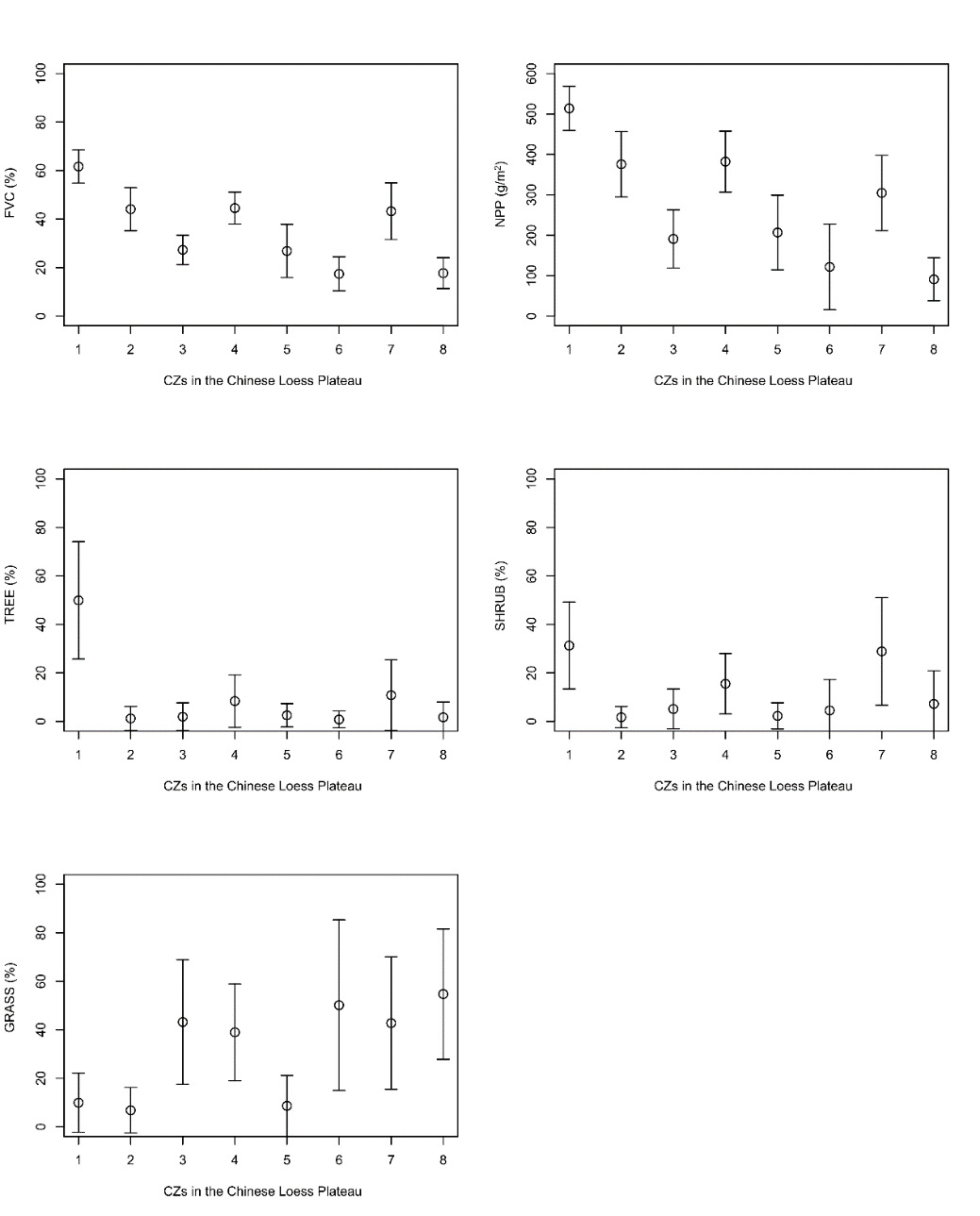


**Supplementary Fig. S18.** Vegetation indicators for the eight CZ classes in the CLP. The sample size (n) of Classes I, II, III, IV, V, VI, VII and VIII are 3870, 2878, 6648, 4868, 229, 4526, 1704 and 4804, respectively.


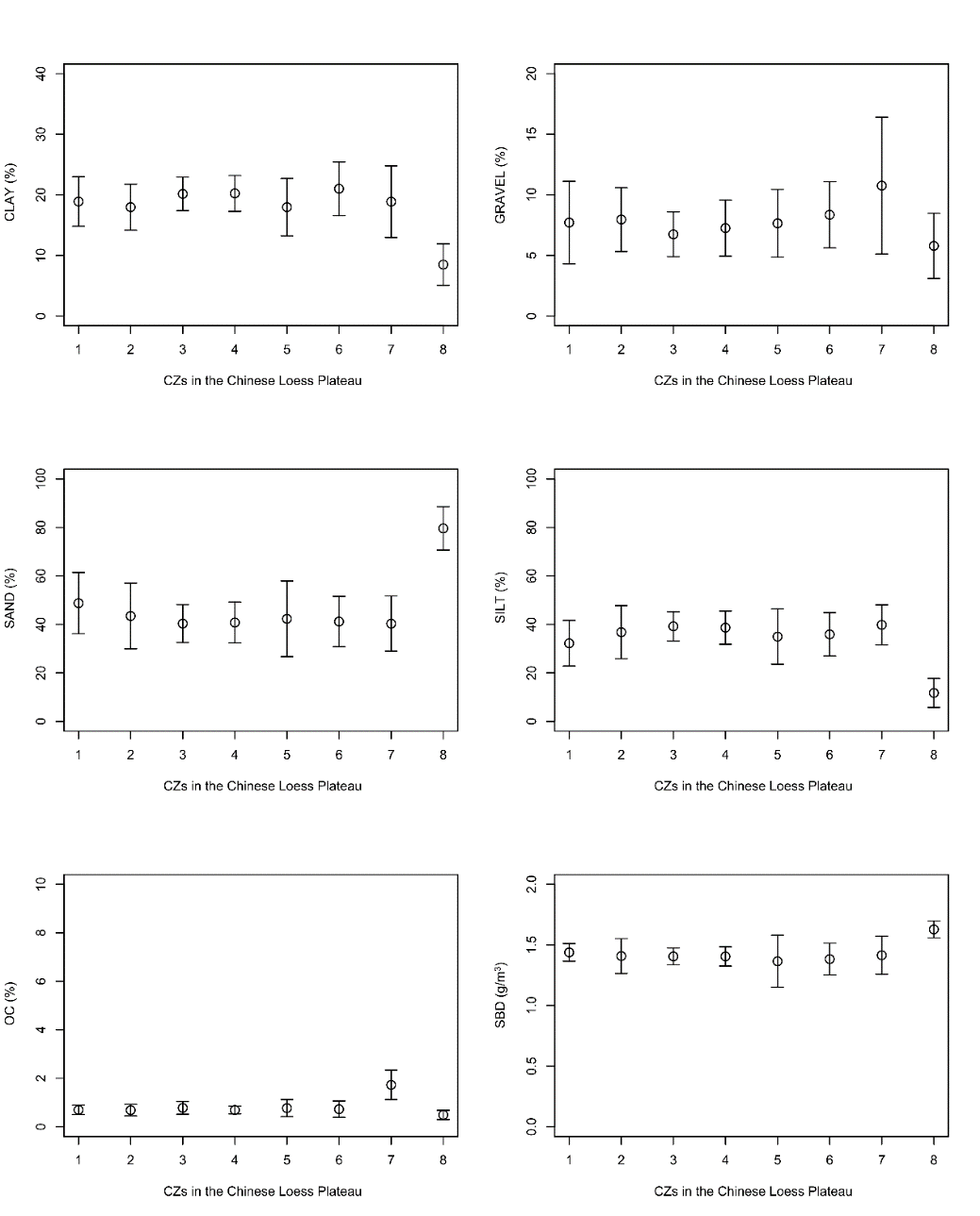


**Supplementary Fig. S19.** Soil properties for the eight CZ classes in the CLP. The sample size (n) of Classes I, II, III, IV, V, VI, VII and VIII are 3870, 2878, 6648, 4868, 229, 4526, 1704 and 4804, respectively.


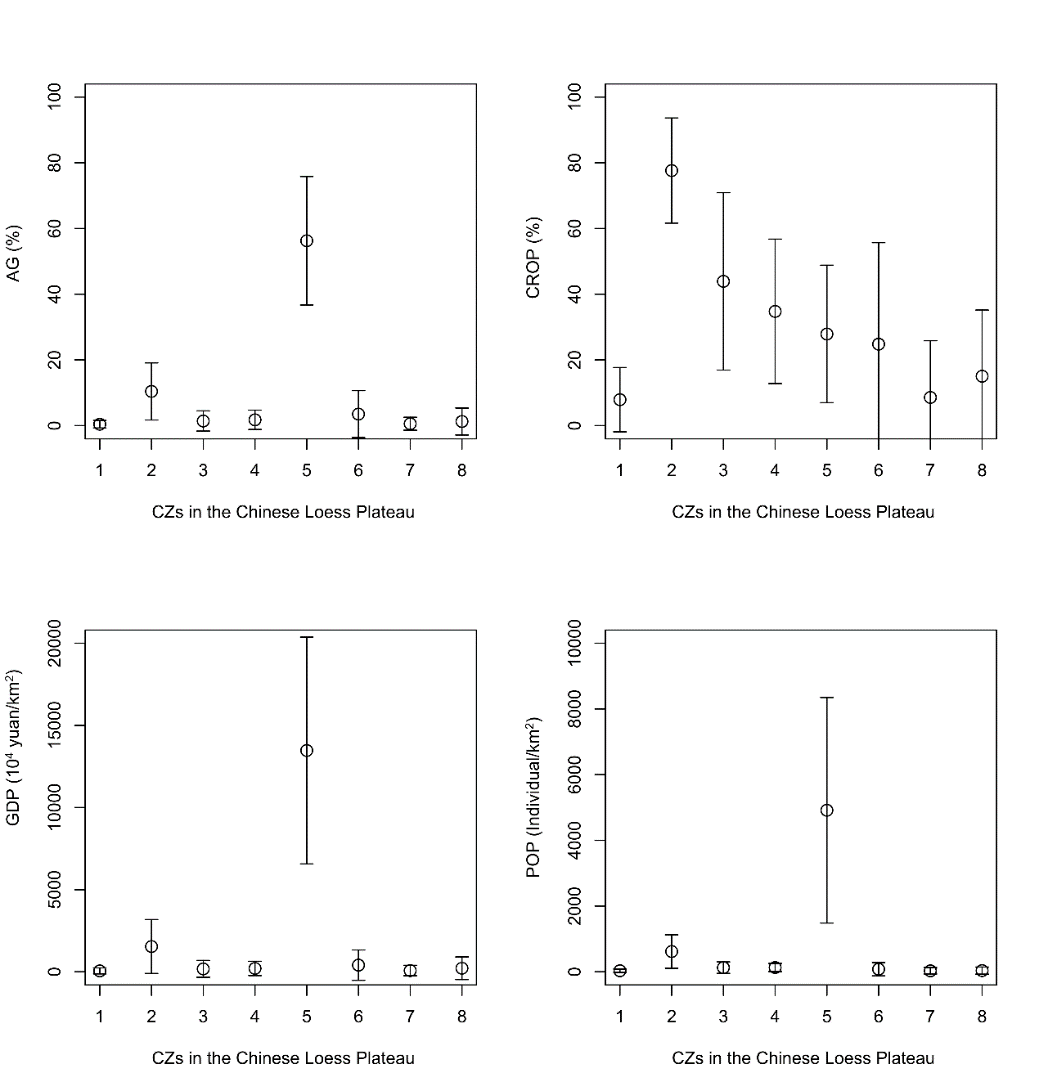


**Supplementary Fig. S20.** Human and socioeconomic indicators for the eight CZ classes in the CLP. The sample size (n) of Classes I, II, III, IV, V, VI, VII and VIII are 3870, 2878, 6648, 4868, 229, 4526, 1704 and 4804, respectively.

**References**

1. Tsunekawa A, Liu G and Yamanaka N *et al.* *Restoration and Development of the Degraded Loess Plateau, China*. Springer Japan, 2014. https://link.springer.com/book/10.1007%2F978-4-431-54481-4.

2. Fu BJ, Wang S and Liu Y *et al.* Hydrogeomorphic Ecosystem Responses to Natural and Anthropogenic Changes in the Loess Plateau of China. *Annu Rev Earth Pl Sc* 2017; **45**: 223-43.

3. Laflen JM. *Soil Erosion and Dryland Farming*. CRC Press, 2000.

4. Lu YH, Zhang LW and Feng XM *et al.* Recent ecological transitions in China: greening, browning, and influential factors. *Sci Rep-Uk* 2015; **5**: 8732.

5. Serrano E and Ruiz-Flaño PAP. Geodiversity: Concept, assessment and territorial application. The case of Tiermes-Caracena (Soria). *Boletín de la AGE* 2007; **45**: 389-94.

6. Serrano E and Ruiz-Flaño PAP. Geodiversity. A theoretical and applied concept. *Geographica Helvetica* 2007; **62**: 8.

7. Benito-Calvo A, Perez-Gonzalez A and Magri O *et al.* Assessing regional geodiversity: the Iberian Peninsula. *Earth Surf Proc Land*. 2009; **34**: 1433-45.

8. Argyriou AV, Sarris A and Teeuw RM. Using geoinformatics and geomorphometrics to quantify the geodiversity of Crete, Greece. *Int J Appl Earth Obs* 2016; **51**: 47-59.

9. Pereira DI, Pereira P and Brilha J *et al.* Geodiversity Assessment of Parana State (Brazil): An Innovative Approach. *Environ Manage* 2013; **52**: 541-52.

10. Silva JP, Pereira DI and Aguiar AM *et al.* Geodiversity assessment of the Xingu drainage basin. *J Maps* 2013; **9**: 254-62.

11. Serrano E and Ruiz-Flaño PAP. Geodiversity assessment in a rural landscape: Tiermes-Caracena Area (Soria, Spain). *Memorie Descrittive Della Carta Geologica D’Italia* 2009; **87**: 8.

12. Hjort J and Luoto M. Geodiversity of high-latitude landscapes in northern Finland. *Geomorphology* 2010; **115**: 109-16.

13. Hjort J and Luoto M. Can geodiversity be predicted from space? *Geomorphology* 2012; **153**: 74-80.

14. Manosso FC and Nóbrega MTd. Calculation of geodiversity from landscape units of the Cadeado Range region in Paraná, Brazil. *Geoheritage* 2015; **8**: 189-99.

15. Ilic MM, Stojkovic S and Rundic L *et al.* Application of the geodiversity index for the assessment of geodiversity in urban areas: an example of the Belgrade city area, Serbia. *Geol Croat* 2016; **69**: 325-36.

16. Ozsahin E. Geodiversity assessment in the Ganos (Isiklar) Mount (NW Turkey). *Environ Earth Sci* 2017; **76**: 271.

17. Gordon JE and Barron HF. The role of geodiversity in delivering ecosystem services and benefits in Scotland. *Scot J Geol* 2013; **49**: 41-58.

18. Gray M, Gordon JE and Brown EJ. Geodiversity and the ecosystem approach: the contribution of geoscience in delivering integrated environmental management. *P Geologist Assoc* 2013; **124**: 659-73.

19. Hjort J, Gordon JE and Gray M *et al.* Why geodiversity matters in valuing nature's stage. *Conserv Biol* 2015; **29**: 630-9.

20. Ke X, Ding XZ and Han KY *et al.* Method for the construction of the geological map database based on MAPGIS-Example from the 1:1M geological map database of China. *Journal of Geomechanics* 2008; **14**: 186-92.

21. Jenness J, Brost B and Beier P. *Land facet corridor designer: Extension for ArcGIS*. Available from: http://wwwjennessentcom/arcgis/land_facetshtm, 2013.

22. Melelli L, Vergari F and Liucci L *et al.* Geomorphodiversity index: Quantifying the diversity of landforms and physical landscape. *Sci Total Environ* 2017; **584**: 701-14.

23. Jenness JS. Calculating landscape surface area from digital elevation models. *Wildlife Soc B* 2004; **32**: 829-39.

24. Weiss A. *Topographic Position and Landforms Analysis*. San Diego, CA: ESRI User Conference, 2001.

25. Feng XM, Fu BJ and Lu N *et al.* How ecological restoration alters ecosystem services: an analysis of carbon sequestration in China's Loess Plateau. *Sci Rep-Uk* 2013; **3**: 2846.

26. Xu XL and Liu W. The global distribution of Earth's critical zone and its controlling factors. *Geophys Res Lett* 2017; **44**: 3201-8.

27. Keith DA. Relationships Between Geodiversity and Vegetation in Southeastern Australia. *P Linn Soc N S W* 2011; **132**: 5-26.

28. Liu Z, Li L and Tim RM *et al.* Introduction of the professional interpolation software for meteorology data: ANUSPLINN. *Meteorological Monthly* 2008; **34**: 92-100.

29. Allen RG, Pereira LS and Raes D *et al*. FAO Penman-Monteith equation. In: *Crop evapotranspiration - Guidelines for computing crop water requirements - FAO Irrigation and drainage paper 56*. Food and Agriculture Organization of the United Nations, 1988.

30. Zapata-Rios X, Brooks PD and Troch PA *et al.* Influence of climate variability on water partitioning and effective energy and mass transfer in a semi-arid critical zone. *Hydrol Earth Syst Sc* 2016; **20**: 1103-15.

31. Rasmussen C, Troch PA and Chorover J *et al.* An open system framework for integrating critical zone structure and function. *Biogeochemistry* 2011; **102**: 15-29.

32. Walker RV and Beck MB. Understanding the metabolism of urban-rural ecosystems A multi-sectoral systems analysis. *Urban Ecosyst* 2012; **15**: 809-48.

33. Rasmussen C and Gallo EL. Technical Note: A comparison of model and empirical measures of catchment-scale effective energy and mass transfer. *Hydrol Earth Syst Sc* 2013; **17**: 3389-95.

34. Rasmussen C, Pelletier JD and Troch PA *et al.* Quantifying Topographic and Vegetation Effects on the Transfer of Energy and Mass to the Critical Zone. *Vadose Zone J* 2015; **14**: vzj2014.07.0102.

35. Troch PA, Lahmers T and Meira A *et al.* Catchment coevolution: A useful framework for improving predictions of hydrological change? *Water Resour Res* 2015; **51**: 4903-22.

36. Rasmussen C. Thermodynamic constraints on effective energy and mass transfer and catchment function. *Hydrol Earth Syst Sc* 2012; **16**: 725-39.

37. Chen Y, Jessel B and Fu BJ *et al.* *Ecosystem Services and Management Strategy in China.* Springer-Verlag Berlin Heidelberg, 2014.

38. Hunsaker CT, Oneill RV and Jackson BL *et al.* Sampling to Characterize Landscape Pattern. *Landscape Ecol* 1994; **9**: 207-26.

39. Birch CPD, Oom SP and Beecham JA. Rectangular and hexagonal grids used for observation, experiment and simulation in ecology. *Ecol Model* 2007; **206**: 347-59.

40. Adamczyk J and Tiede D. ZonalMetrics - a Python toolbox for zonal landscape structure analysis. *Comput Geosci-Uk* 2017; **99**: 91-9.

41. Wu NN, Yan XP and Huang GH *et al.* Urban environment-oriented traffic zoning based on spatial cluster analysis. *J Environ Inform* 2010; **15**: 111-9.

42. Hartigan JA and Wong MA. A K-means clustering algorithm. *Appl Stat* 1979; **28**: 100-8.

43. Kabacoff RI. *R in Action: Data analysis and graphics with R*. Shelter Island, New York: Manning Publications, 2015.
